# Hypochlorous Acid-Responsive Prodrug Nanoplatform for Synergistic Cancer Immunotherapy

**DOI:** 10.34133/bmr.0300

**Published:** 2026-01-23

**Authors:** Shu Xia, Xinyu Wang, Cheng Liu, Ran Ji, Mingzhi Wang, Chi Zhang, Liang Chen, Wenqiang Chen, Shao Q. Yao, Chao Fang, Xiao Dong

**Affiliations:** ^1^ Shanghai 411 Hospital, China RongTong Medical Healthcare Group Co. Ltd./411 Hospital, Shanghai University, Shanghai 200081, China.; ^2^ Institute of Artificial Intelligence and Biomanufacturing, School of Medicine, Shanghai University, Shanghai 200444, China.; ^3^ Shanghai Tenth People’s Hospital of Tongji University, Shanghai 200072, China.; ^4^ Guangxi Key Laboratory of Natural Polymer Chemistry and Physics, College of Chemistry and Materials Science, Nanning Normal University, Nanning 530001, China.; ^5^ Department of Chemistry, National University of Singapore, Singapore 117543, Singapore.; ^6^ Hongqiao International Institute of Medicine, Tongren Hospital and State Key Laboratory of Systems Medicine for Cancer, Shanghai Jiao Tong University School of Medicine, Shanghai 200025, China.

## Abstract

Immunotherapy offers a promising paradigm for cancer treatment, but its efficacy is often constrained by tumor heterogeneity and the immunosuppressive tumor microenvironment. Herein, we constructed a multifunctional nanoplatform (termed MD1a NP) designed to elicit personalized antitumor immunity and overcome tumor immunosuppression by co-assembling a hypochlorous acid (HOCl)-responsive methylene blue (MB)–doxorubicin (DOX) dimer prodrug with a stimulator of interferon genes (STING) agonist (1a). Following intravenous administration, elevated intratumoral HOCl triggers the activation and release of MB and DOX, inducing nanoparticle disassembly and facilitating the liberation of 1a. Upon near-infrared laser irradiation, MB-mediated photodynamic therapy synergizes with DOX-induced chemotherapy to eradicate tumor cells and amplify immunogenic cell death, thereby enhancing the release of tumor antigens and damage-associated molecular patterns. This cascade promotes dendritic cell maturation, which is further reinforced by 1a-mediated STING activation. Moreover, MD1a NP treatment decreases regulatory T-cell populations, alleviates T-cell suppression, and promotes memory T-cell formation. Consequently, MD1a NP combined with laser irradiation remodels the immunosuppressive tumor microenvironment and effectively inhibits both primary and distant tumor growth while preventing lung metastasis in orthotopic 4T1 breast cancer models. This study provides insights into the design of tumor-activatable nanoplatforms for multimodal therapy against immune-desert cancers.

## Introduction

Immunotherapy, which leverages the host immune system to selectively attack cancer cells, offers improved outcomes compared to traditional treatment modalities such as surgery, chemotherapy, and radiotherapy [1]. However, a subset of patients fails to respond to immunotherapies such as immune checkpoint inhibitors, immune adjuvants, and therapeutic cancer vaccines. Solid tumors often develop a microenvironment characterized by low immunogenicity and the presence of immunosuppressive cytokines and cells, which severely impair antitumor immune processes, including dendritic cell (DC) maturation, antigen presentation, and T-cell-mediated tumor elimination [2,3]. Moreover, genetic and epigenetic alterations induced by immunotherapies, together with immunosuppressive signaling pathways such as transforming growth factor-β and programmed cell death protein 1/programmed cell death ligand 1, enable cancer cells to evade immune surveillance [4,5]. Tumor heterogeneity represents another major barrier to effective treatment responses, underscoring the need to elicit personalized antitumor immunity. Collectively, these challenges highlight the urgent need for combination strategies that enhance tumor antigen presentation, remodel the immunosuppressive tumor microenvironment (TME), and overcome tumor heterogeneity.

Using autologous tumor cells, which contain a broad spectrum of tumor antigen epitopes, to prepare in situ tumor vaccination (ISTV) offers a simple and effective strategy for eliciting personalized and broad-spectrum antitumor immunity. Clinical modalities such as chemotherapy, radiotherapy, and photodynamic therapy (PDT) not only inhibit tumor progression but also induce immunogenic cell death (ICD), thereby facilitating localized exposure of tumor antigens and damage-associated molecular patterns (DAMPs) that activate both innate and adaptive immune responses [6–12]. Notably, combining 2 mechanistically distinct ICD inducers, such as PDT and chemotherapy, can synergistically amplify ICD and achieve superior therapeutic efficacy compared with monotherapy. Nevertheless, ICD inducers alone often show limited efficacy in evoking robust antitumor immunity, likely due to impaired DC functionality and insufficient infiltration of cytotoxic T lymphocytes (CTLs) within the immunosuppressive TME [13–15]. This limitation highlights the critical role of immunological adjuvants in remodeling the TME into an immune-activating milieu that supports DC maturation and T-cell function [16–18]. Activation of the stimulator of interferon genes (STING) pathway promotes the secretion of pro-inflammatory cytokines, including type I interferons (IFNs), tumor necrosis factor-α (TNF-α), and interleukin-6 (IL-6) [19–22]. These cytokines enhance DC maturation and antigen-presenting capability, thereby facilitating the priming and intratumoral infiltration of CTLs [21,23]. Therefore, the combination of ICD induction with immune adjuvants provides a promising strategy for eliciting potent antitumor immunity. Importantly, the development of novel pharmaceutical approaches that enable tumor-selective accumulation and activation of ICD inducers or immune adjuvants is essential to ensure both the efficacy and safety of ISTV [24].

**Fig. 1.**
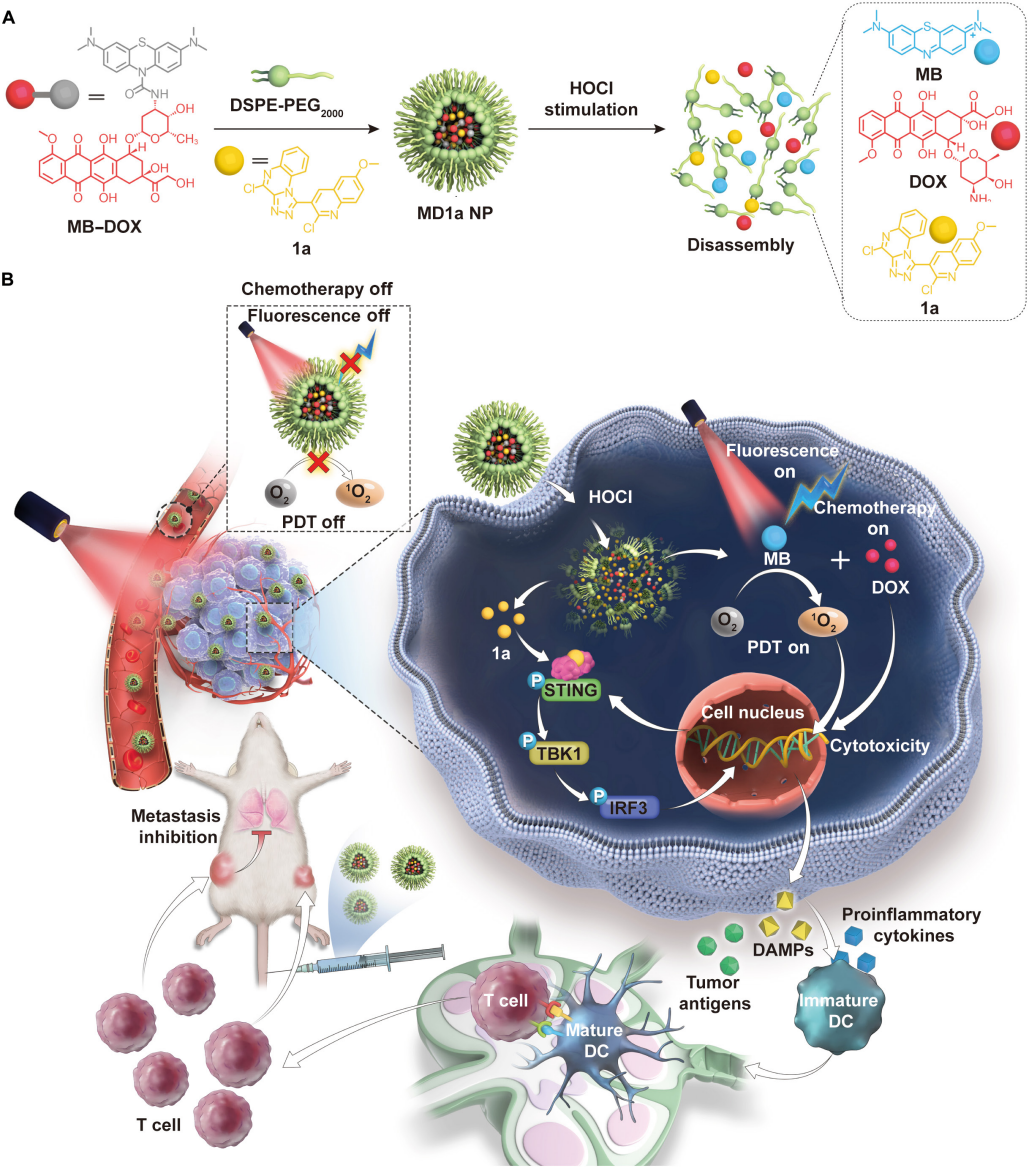
Schematic illustration of the design and mechanism of MD1a NP for synergistic cancer immunotherapy. (A) The HOCl-responsive methylene blue (MB)–doxorubicin (DOX) dimer prodrug co-assembles with the stimulator of interferon genes (STING) agonist 1a to form a tumor-activatable nanoplatform (MD1a NP). HOCl stimulation activates and triggers the release of MB and DOX, which in turn promotes nanoparticle disassembly and facilitates the subsequent liberation of 1a. (B) Following intravenous administration, elevated intratumoral HOCl triggers the activation and the subsequent release of MB and DOX, enabling synergistic photochemotherapy under near-infrared (NIR) laser irradiation. This process induces robust immunogenic cell death (ICD) while minimizing systemic off-target effects. Concurrently, HOCl-triggered nanoparticle disassembly accelerates 1a release, activating the STING pathway and establishing an immune-promoting tumor microenvironment (TME). In orthotopic 4T1 breast cancer mouse models, MD1a NP-mediated in situ tumor vaccination (ISTV) elicited strong antitumor immunity, effectively inhibiting both primary and distant tumor growth, preventing lung metastasis, and prolonging overall survival. PDT, photodynamic therapy; DAMPs, damage-associated molecular patterns; DC, dendritic cell.

Activatable nanomedicines that enable tumor-selective drug release and enhance drug bioavailability provide a promising platform for achieving effective ISTV. For example, stimulus-responsive prodrug nanomedicines that respond to exogenous stimuli (e.g., light or ultrasound) or abnormal pathological features of tumors (e.g., glutathione and reactive oxygen species) have been developed to minimize the off-target side effects of ICD inducers and immune adjuvants [8,25–28]. Elevated levels of hypochlorous acid (HOCl) in tumors, compared with those in normal tissues, provide a promising endogenous stimulus for tumor-specific drug activation [29–31]. To achieve efficient ISTV by synergizing photochemotherapy-induced ICD with immune adjuvants while minimizing systemic toxicity, we developed a HOCl-responsive dimeric prodrug by conjugating doxorubicin (DOX) to leucomethylene chloride (FDOCl-2), a derivative of the photosensitizer methylene blue (MB). The resulting MB–DOX prodrug co-assembled with a nonnucleoside STING agonist (1a) into a multifunctional nanoplatform (MD1a NP) through hydrophobic interactions and π–π stacking (Fig. 1A). Following intravenous administration, elevated HOCl levels in tumors trigger the activation and release of MB and DOX. This design prevents the “always-on” activity of the therapeutic agents during circulation and mitigates the reduced efficacy associated with their differential pharmacokinetic profiles. Upon near-infrared (NIR) laser irradiation, MB-mediated PDT synergizes with DOX-induced chemotherapy to kill cancer cells and induce potent ICD, thereby promoting the release of DAMPs and tumor antigens (Fig. 1B). Meanwhile, HOCl-triggered nanoparticle disassembly facilitates the release of 1a at tumor sites, thereby activating the STING pathway to enhance DC maturation and antigen presentation, as well as to promote the priming and intratumoral infiltration of CTLs. In orthotopic 4T1 breast cancer models, treatment with MD1a NP combined with NIR irradiation effectively reduced the proportion of regulatory T cells (Tregs), alleviated T-cell immunosuppression, and promoted the formation of memory T cells. Collectively, these effects reversed the immunosuppressive TME and elicited systemic antitumor immunity, thereby inhibiting both primary and abscopal tumor growth, preventing lung metastasis, and prolonging overall survival (Fig. 1B). This study introduces a promising approach for developing a stimulus-responsive nanoplatform for multimodal therapy against immune-desert tumors.

## Materials and Methods

### Materials

All chemical reagents were acquired from Sigma-Aldrich, USA, unless otherwise specified. All solvents were obtained from Sinopharm Chemical Reagent Co., Ltd. STING agonist 1a (HY-131994) and Cell Counting Kit-8 (CCK-8) were purchased from MedChemExpress, China. DSPE-mPEG (molecular weight 2,000 Da) and doxorubicin hydrochloride were purchased from Shanghai Aladdin Biochemical Technology Co., Ltd., China. Singlet Oxygen Sensor Green (SOSG) fluorescent probe, 2′,7′-dichlorodihydrofluorescein diacetate (DCFH-DA), cytokine (TNF-α, interferon-beta [IFN-β], and IL-6) assay kit, adenosine triphosphate (ATP) assay kit, 4′,6-diamidino-2-phenylindole (DAPI), protease inhibitor, rabbit anti-mouse calreticulin (CRT) monoclonal antibody (mAb), Alexa Fluor 555-labeled donkey anti-rabbit IgG(H+L), rabbit anti-mouse β-actin mAb, and horseradish peroxidase (HRP)-labeled goat anti-rabbit IgG(H+L) were purchased from Beyotime Biotech Inc., Shanghai, China. HMGB1 assay kit, GM-CSF, and IL-6 were supplied by ABclonal Technology Co., Ltd., Wuhan, China. Phospho-STING (Ser365) (D8F4W) rabbit mAb (no. 72971), phospho-IRF3 (Ser379) (E6F7Q) rabbit mAb (no. 79945), and phospho-TBK1/NAK (Ser172) (D52C2) XP rabbit mAb (no. 5483) were purchased from Cell Signaling Technology, Inc. Phycoerythrin (PE) Armenian hamster anti-mouse CD11c (N418), Ms CD80 allophycocyanin (APC) 16-10A1, Brilliant Violet 421 (BV421) rat anti-mouse CD86(GL1), CD3 (17A2) rat mAb (fluorescein isothiocyanate [FITC] conjugate), and APC rat anti-mouse CD4 (RM4-5) were purchased from BD Pharmingen. Mouse CD8 alpha PE-conjugated antibody was purchased from R&D Systems. Fetal bovine serum (FBS), Dulbecco’s modified Eagle medium (DMEM), penicillin, streptomycin, and trypsin were purchased from Thermo Fisher Scientific Inc. Ultrapure water was obtained using a Millipore Milli-Q system (Bedford, MA).

The murine breast cancer cell line 4T1 and mouse fibroblast cell line NIH 3T3 were cultured in DMEM supplemented with penicillin (100 U ml^−1^), streptomycin (100 μg ml^−1^), and 10% FBS. Cells were incubated in a cell incubator at 37 °C under a 5% CO_2_ atmosphere. BALB/c mice (female, 6 to 7 weeks old) were purchased from Jiangsu Huachuang Xina Pharmaceutical Technology Co. Ltd, China. All animal-related experiments were conducted according to the ethical policies and procedures approved by the ethics committee of the School of Medicine, Shanghai University, China (Approval No. ECSHU 2022-105).

### Synthesis of FDOCl-2

The synthesis of FDOCl-2 was carried out according to a published protocol [29]. To a solution of MB (2.5 g, 7.82 mmol, 1.0 eq) in 50 ml of water, sodium carbonate (4.97 g, 46.89 mmol, 8.0 eq) and 15 ml of dichloromethane were added. The resulting mixture was warmed to 45 °C and stirred under a nitrogen atmosphere for 5 min. Subsequently, 50 ml of aqueous solution of sodium dithionite (10.88 g, 62.52 mmol, 4.0 eq) was injected directly into the above solution through a syringe. The reaction mixture was continuously agitated under a nitrogen atmosphere until the solution became yellow and then cooled to 4 °C with an ice-water bath, and bis(trichloromethyl) carbonate (2.32 g, 7.82 mmol, 0.5 eq) dissolved in 15 ml of dichloromethane was added dropwise. After that, the reaction mixture was further stirred for an additional 1 h, poured into 150 ml of ice-cold water, and extracted by dichloromethane (100 ml × 3 times) The combined organic phase was washed successively with brine and water and dried over anhydrous sodium sulfate. After removal of the solvent under reduced pressure, the residue was further purified by flash chromatography (using ethyl acetate/*n*-hexane = 1:8 as the eluent) to give FDOCl-2 (1.28 g, yield 46%) as a white solid.

### Synthesis of MB–DOX

To a solution of FDOCl-2 (1 g, 2.87 mmol, 1.0 eq) in 10 ml of dichloromethane, sodium carbonate (0.61 g, 5.74 mmol, 3.0 eq) was added. Subsequently, a solution of DOX (1.87 g, 11.48 mmol, 4.0 eq) in 20 ml of dichloromethane was added dropwise, and the obtained reaction mixture was stirred at room temperature for 2 h. After quenching the reaction by adding 150 ml of ice water, the resulting mixture was extracted with dichloromethane (100 ml 3 times). The combined organic phase was washed successively with brine and water and dried over anhydrous sodium sulfate. After removal of the solvent under reduced pressure, the oily residue was further purified by flash chromatography (using ethanol/dichloromethane = 1:80 as the eluent) to give MB–DOX (1.05 g, yield 43%) as an orange solid.

### Selectivity of MB–DOX toward HOCl

Various ROS and reactive nitrogen species (RNS) were initially prepared in distilled water. Diluted H_2_O_2_ solution was derived from a 30% stock solution. KO_2_ was dissolved in dimethyl sulfoxide (DMSO) to generate O_2_^−^. ·OH was produced via the Fenton reaction by adding H_2_O_2_ in the presence of 4 to 10 equivalents of iron(II) sulfate heptahydrate, achieving a concentration of ·OH equivalent to that of H_2_O_2_. ONOO^−^ was synthesized using 3-morpholinosydnonimine hydrochloride. Subsequently, a stock solution of MB–DOX in DMSO at 1 mM was diluted to a final concentration of 20 μM in sodium phosphate buffer (PBS; pH 7.4) supplemented with the various ROS and RNS. The absorption and fluorescence spectra of the solution were recorded using an ultraviolet–visible (UV–vis) spectrometer (UV-1800, Shimadzu) and a Fluorolog 3-TCSPC spectrofluorometer (Horiba Jobin Yvon), respectively. Absorption measurements were conducted over a wavelength range of 400 to 800 nm, while fluorescence spectra were acquired with an excitation wavelength of 620 nm, detecting emissions within the range of 630 to 800 nm.

### Preparation and characterization of MD1a NP

MB–DOX, DSPE-PEG_2000_, and 1a were dissolved in DMSO to prepare a stock solution (10 mg/ml). MD1a NP was fabricated following previously reported procedures with minimal modifications [32]. Specifically, 13.3 μl of the MB–DOX stock solution, 7 μl of the 1a stock solution, and 6 μl of the DSPE-mPEG_2000_ stock solution were combined in 200 μl of DMSO. The mixture was rapidly added to 2 ml of PBS while stirring at 1,200 rpm for 2 h. The resulting formulation underwent overnight dialysis in PBS using a dialysis membrane (molecular weight cutoff [MWCO] 10 kDa) to remove free drugs and residual organic solvent. The fabrication of MD NP followed the same procedures, excluding the addition of 1a. The hydrodynamic sizes and zeta potentials of prodrug nanoassemblies were characterized by dynamic light scattering (DLS) using Zetasizer Pro (Malvern). The shape and morphology of the prodrug nanoassemblies were characterized with a JEM-1400Flash electron microscope. Changes in the absorption and fluorescence spectra of MD1a NP in response to HOCl was investigated using the aforementioned methodology. To study the colloidal stability of the prodrug nanoassemblies, MD1a NP or MD NP was dispersed in PBS supplemented with 10% FBS and shaken at 200 rpm at 37 °C. The hydrodynamic sizes of the nanoparticles were measured by DLS at various time points.

### Encapsulation efficiency (EE%) and drug-loading efficiency (DLE%)

The EE% of MB–DOX and 1a within MD1a NP was determined by calculating the percentage of the remaining drug after dialysis, utilizing an ultrafiltration tube (MWCO 100 kDa). Briefly, 2 ml of freshly prepared MD1a NP was placed in the upper chamber of an Amicon Ultra centrifugal filter and centrifuged at 3,000 g for 5 min. The MD1a NP retained in the upper chamber was then resuspended in the original volume of PBS and subjected to 3 consecutive centrifugation cycles. The filtrates were combined, and the concentrations of drugs in these filtrates were analyzed using high-performance liquid chromatography (HPLC), with free drugs serving as standards for quantification. EE% was calculated as in the following equation: EE% = ((total mass of drug during nanoparticle preparation − mass of free drug in filtrates)/(total mass of drug during nanoparticle preparation)) × 100%. To determine the DLE% of MB–DOX and 1a within MD1a NP, 2 mg of the lyophilized MD1a NP after purification was dissolved in DMSO. The masses of MB–DOX and 1a were determined using HPLC. DLE% was calculated using the equation DLE% = (mass of drug in MD1a NP/mass of MD1a NP) × 100%.

### HOCl-triggered drug release

MD1a NP was dispersed in PBS (pH 7.4) supplemented with varying HOCl concentrations (0 to 400 μM) and incubated at 37 °C for 10 min. Subsequently, the MD1a NP solution was transferred to the upper chamber of an ultrafiltration tube (MWCO 100 kDa) and centrifuged at 3,000 g for 5 min. The concentrations of the released MB, 1a, and DOX in the filtrates were quantified using HPLC, with standard curves established using solutions of the individual free drugs. An Agilent HC-C18 column was employed for the HPLC analysis, utilizing a mobile phase consisting of methanol and distilled water, with the addition of 0.1% formic acid to enhance analytical efficiency. Data acquisition and analysis were performed at wavelengths of 254 nm for 1a, 484 nm for MB, and 660 nm for DOX.

### In vitro ROS generation and detection

SOSG was employed to evaluate the capability of MD1a NP to generate ROS in vitro. MD1a NP (with an equivalent MB–DOX concentration of 100 μM) was dispersed in PBS containing 10 μM SOSG and varying HOCl concentrations (0, 5, 10, 20, 50, and 100 μM). The solution was then irradiated with a 660-nm laser (400 mW/cm^2^) for 2 min. Changes in SOSG fluorescence (Ex/Em = 488/525 nm) were promptly measured.

### Hemolysis test

Fresh mouse blood was collected and placed in heparinized tubes at 4 °C. To separate plasma from erythrocytes, the collected blood was centrifuged at 3,000 rpm for 5 min. The isolated erythrocytes were then washed with PBS and subjected to 3 additional rounds of centrifugation (3,000 rpm, 5 min) to remove the supernatant. Subsequently, the erythrocyte suspension was supplemented with MD1a NP at concentrations ranging from 0 to 0.8 mg/ml while maintaining a constant erythrocyte concentration of 5%. After incubating at 37 °C for 1 h, the mixture was centrifuged at 3,000 rpm for 5 min. The concentration of released hemoglobin in the supernatant was quantified using UV–vis spectroscopy at a wavelength of 541 nm. The MD1a NP solution at the corresponding concentration was centrifuged at 3,000 rpm for 5 min, and the resulting supernatant was used as the background control. To prepare the positive control, the erythrocyte suspension was diluted in distilled water to a 5% concentration. In contrast, the negative control was prepared by diluting erythrocytes in PBS to a 5% concentration without the addition of MD1a NP. The percentage of hemolysis was calculated using the following formula: Hemolysis (%) = ((absorbance of sample − absorbance of background − absorbance of negative control)/(absorbance of positive control − absorbance of negative control)) × 100%.

### In vitro selective cancer cell imaging

The capacity of MD1a NP for selective imaging of cancer cells was evaluated using flow cytometric assay and confocal laser scanning microscopy (CLSM). For the flow cytometric assay, NIH 3T3 cells and 4T1 cells were separately seeded into 24-well microplates (4 × 10^4^ cells per well) and cultured in DMEM at 37 °C with 5% CO_2_ for 12 h. Following treatment with MD1a NP (with an equivalent MB–DOX concentration of 10 μM) for 6 h, both cell types were collected, washed with PBS, and analyzed using flow cytometry. For CLSM imaging, NIH 3T3 cells and 4T1 cells were seeded onto 14-mm glass coverslips in 24-well microplates (4 × 10^4^ cells per well) and incubated for an additional 12 h. After treatment with MD1a NP (at an equivalent MB–DOX concentration of 10 μM) for 6 h, the cells were washed with PBS and fixed in 4% paraformaldehyde at room temperature for 5 min. Subsequently, both cell types were stained with DAPI and observed using CLSM.

### In vitro cytotoxicity

The cytotoxicity of MD1a NP on 4T1 and NIH 3T3 cells was evaluated using CCK-8 assay kits. Initially, 4T1 and NIH 3T3 cells were seeded into a 96-well plate (1 × 10^4^ cells per well) and incubated at 37 °C with 5% CO_2_ for 24 h. Following incubation with various concentrations of MD1a NP for an additional 6 h, the cells were washed 3 times with PBS and subjected to laser irradiation (660 nm, 400 mW/cm^2^, 2 min) per well. Subsequently, the cells were incubated with 100 μl of DMEM containing 10% CCK-8 for 2 h at 37 °C under 5% CO_2_, and the absorbance of the solution was measured using a microplate reader at 450 nm.

### Intracellular ROS generation

4T1 and NIH 3T3 cells were seeded onto 14-mm glass coverslips (5 × 10^4^ cells per well) in a 24-well plate and cultured for an additional 12 h. The cells were treated with MD1a NP at an equivalent MB–DOX concentration of 10 μM for 4 h, followed by incubation with fresh DMEM containing DCFH-DA (10 μM) for 1 h. After washing with PBS, the cells were exposed to NIR laser irradiation (660 nm, 400 mW/cm^2^, 2 min). Subsequently, 4T1 cells were fixed in 4% paraformaldehyde at room temperature for 5 min. Finally, 4T1 cells were stained with DAPI and examined using CLSM to assess the intracellular levels of ROS.

### Evaluation of MD1a NP-induced ICD

Briefly, 4T1 cells were seeded onto 14-mm glass coverslips in a 24-well plate (5 × 10^4^ cells per well) and incubated for 24 h. Subsequently, the cells were treated with either PBS, DOX, MB (+), MD1a NP, MD NP (+), or MD1a NP (+) for 12 h. The MB–DOX equivalent dose was 10 μM. Following treatment, the cells receiving NIR laser treatment were washed with PBS, subjected to NIR laser irradiation (660 nm, 400 mW/cm^2^, 2 min), and then cultured for an additional 12 h. The concentrations of ATP and HMGB1 released into the culture medium were evaluated using an ATP assay kit and an HMGB1 enzyme-linked immunosorbent assay (ELISA) kit, respectively. To investigate the dislocation of CRT in 4T1 cells, the cells subjected to different treatments were blocked with 5% bovine serum albumin (BSA) in PBS for 10 min and then incubated with rabbit anti-mouse CRT mAb overnight at 4 °C. The surface expression of CRT on 4T1 cells was evaluated using CLSM after staining with Alexa Fluor 555-labeled donkey anti-rabbit IgG(H+L) and DAPI.

### In vitro STING activation

The assessment of key proteins associated with the activation of the STING pathway was conducted using Western blotting. Briefly, 4T1 cells were treated with either PBS, DOX, MB (+), MD1a NP, MD NP (+), or MD1a NP (+) for 12 h, at an equivalent MB–DOX concentration of 10 μM. The 4T1 cells that received NIR laser treatment were washed with PBS and subjected to NIR laser irradiation (660 nm, 400 mW/cm^2^, 2 min). After incubating for an additional 24 h, the 4T1 cells were lysed on ice using radioimmunoprecipitation assay buffer supplemented with protease inhibitors to extract proteins. Protein concentrations were determined using a bicinchoninic acid assay kit. Equal amounts of protein were separated by 10% sodium dodecyl sulfate–polyacrylamide gel electrophoresis, transferred to a polyvinylidene difluoride (PVDF) membrane, and blocked with Tris-buffered saline solution containing 5% BSA for 1.5 h. The PVDF membranes were then incubated with primary antibodies targeting key proteins, specifically p-STING, p-IRF3, and p-TBK1, at 4 °C overnight. Chemiluminescence detection using an enhanced chemiluminescence substrate was performed on the PVDF membranes after incubation with HRP-labeled goat anti-rabbit secondary antibody.

### BMDC differentiation

Bone-marrow-derived monocytes (BMDMs) were obtained from the mouse femur and tibia using a previously reported method [33]. The harvested BMDMs were cultured in RPMI 1640 medium supplemented with 10% FBS and 1% penicillin–streptomycin at 37 °C under 5% CO_2_. Bone-marrow-derived dendritic cells (BMDCs) were differentiated by adding recombinant mouse GM-CSF (20 ng/ml) and IL-4 (10 ng/ml) to the medium every 2 d. After 7 d, the BMDCs were harvested for further use.

### In vitro pro-inflammatory cytokine secretion and DC maturation

A transwell co-culture system was employed to evaluate the impact of MD1a NP-induced ICD on DC maturation in vitro. Briefly, 4T1 cells were pre-treated with either PBS, DOX, MB (+), MD1a NP, MD NP (+), or MD1a NP (+) for 12 h, with an equivalent MB–DOX concentration of 10 μM. The 4T1 cells receiving NIR laser treatment were washed with PBS and subjected to NIR laser irradiation (660 nm, 400 mW/cm^2^, 2 min). Subsequently, the 4T1 cells were seeded into the upper chamber to incubate with the harvested BMDCs seeded into the lower chamber. After co-incubating for 36 h, the pro-inflammatory cytokines (such as TNF-α, IFN-β, and IL-6) released into the culture medium were evaluated using ELISA kits. The maturation of DCs in the lower chamber was analyzed using flow cytometry following staining with anti-mouse CD11c, anti-mouse CD80, and anti-mouse CD86.

### In vivo selective tumor imaging of MD1a NP

To investigate the ability of MD1a NP for selective tumor imaging in vivo, an orthotopic 4T1 breast cancer mouse model was established by injecting 4T1 cells into the breast fat pad. Once the tumor volume reached 300 mm^3^, MD1a NP was intravenously injected into the mice at an equivalent MB dose of 1.4 mg/kg, with free MB serving as the control group. Fluorescence imaging was conducted at 4, 8, and 12 h postinjection using the IVIS Spectrum system, with an excitation wavelength of 660 nm and an emission wavelength of 700 nm. To evaluate the in vivo biodistribution of MD1a NP and MB, the mice were euthanized 12 h postinjection, and both tumors and major organs (including the heart, liver, spleen, lungs, and kidneys) were excised for ex vivo imaging using the IVIS Spectrum system.

### Detection of HOCl levels in tissues

An orthotopic 4T1 breast tumor-bearing mouse model was established. Mice were intravenously injected with the HOCl-specific fluorescent probe EtS-DMAB at 5 mg/kg. One hour postinjection, the mice were euthanized, and tumors along with major organs were immediately harvested. The samples were embedded in OCT compound and rapidly frozen. Frozen tissues were sectioned into 10-μm-thick slices and fixed with 4% paraformaldehyde for 10 min. The nuclei were then counterstained with DAPI for 10 min. Finally, the sections were mounted using an anti-fade medium, and fluorescence images were acquired using CLSM.

### In vivo antitumor therapy

The orthotopic 4T1 breast cancer model, established as described above, was used to evaluate the antitumor efficacy of MD1a NP. When the tumor volume reached approximately 150 mm^3^, mice were randomly divided into 6 groups (*n* = 5) as follows: G1, PBS; G2, MB (+); G3, DOX; G4, MD1a NP; G5, MD NP (+); and G6, MD1a NP (+). Various formulations were intravenously injected 4 times every 2 d, with equivalent doses of MB–DOX and 1a set at 10 and 4.3 mg/kg, respectively. Mice in the MB (+), MD NP (+), and MD1a NP (+) treatment groups received NIR laser irradiation (660 nm, 500 mW/cm^2^, 2 min) 4 h posttreatment. The tumor volume and body weight of the mice were monitored every 2 d, with tumor volume calculated using the formula *V* = (length × width^2^)/2. On day 15, the mice were euthanized, and both tumors and major organs were harvested for further analysis. Tumor samples were subjected to histological evaluation using hematoxylin and eosin (H&E) staining to assess tissue morphology. Apoptotic cells and cellular proliferation within the tumor tissues were identified through immunofluorescence staining using terminal deoxynucleotidyl transferase-mediated dUTP nick-end labeling (TUNEL) markers, respectively. Additionally, to evaluate the in vivo safety profile of MD1a NP, key organs were analyzed histologically using H&E staining. To evaluate the inhibitory effect of MD1a NP on tumor lung metastasis, tumor-bearing mice were subjected to different treatments. On day 30, the mice were euthanized, and their lungs were harvested and immersed in PBS. Images of the lungs were acquired, and surface metastatic nodules were counted to quantify metastatic burden. The lung tissues were then fixed in 4% paraformaldehyde and subjected to histological analysis of metastatic lesions using H&E staining.

### Mechanisms of MD1a NP-mediated antitumor immunity

To elucidate the mechanisms underlying MD1a NP-induced antitumor immune responses, tumors were harvested from mice on day 15 and prepared into single-cell suspensions for immune cell phenotype analysis via flow cytometry. Tumor tissues were first dissected into small fragments and then enzymatically digested with a mixture containing hyaluronidase (1.5 mg/ml), collagenase I (1.5 mg/ml), collagenase IV (1.5 mg/ml), and DNase I (0.2 mg/ml) in DMEM at 37 °C for 1 h. Following digestion, the cell suspensions are treated with red blood cell lysis buffer and filtered through a 70-μm cell filter 3 times. The filtered cell suspensions were then washed 3 times with PBS in preparation for further analysis. The percentage of DC maturation in tumor tissues was assessed by flow cytometry following staining with PE-conjugated anti-mouse CD11c, APC-conjugated anti-mouse CD80, and BV421-conjugated anti-mouse CD86 antibodies. The activation of cytotoxic T cells within the tumor tissues was assessed through flow cytometry after staining with FITC-labeled CD3 (17A2) rat mAb, APC-conjugated rat anti-mouse CD4 (RM4-5), and PE-conjugated mouse CD8 alpha antibody. Additionally, tumor tissues were subjected to immunohistochemical (IHC) staining utilizing CD8 as a marker to evaluate the tumor infiltration of CTLs.

### In vivo ICD detection

The synergistic effects of photochemotherapy on inducing ICD in vivo were evaluated by analyzing the exposure of CRT and the release of HMGB1 and ATP in tumor tissues. Immunofluorescence staining was employed to assess intratumoral CRT exposure. Briefly, tumor tissues were fixed in paraformaldehyde and then frozen and sectioned. After being blocked with PBS supplemented with 5% BSA, the resulting tissue sections were incubated with primary antibodies against CRT. Imaging was performed using CLSM after staining with fluorescent-dye-conjugated secondary antibodies and DAPI. Additionally, the HMGB1 and ATP levels in tumor tissue homogenates were measured using an HMGB1 ELISA kit and an ATP assay kit, respectively, following the manufacturer’s instructions, to determine intratumoral HMGB1 and ATP release across different experimental groups.

### Assessment of the abscopal therapeutic effect and underlying mechanism

To investigate the abscopal therapeutic effect of MD1a NP, a bilateral 4T1 tumor model was established by injecting 4T1 cells into the left breast fat pad (1 × 10^6^ cells) and the right breast fat pad (5 × 10^5^ cells), designating the left tumor as the primary tumor and the right tumor as the distant tumor. Once the tumor volume reached approximately 50 mm^3^, the bilateral tumor-bearing mice were randomly assigned to 6 groups (*n* = 5). The primary 4T1 tumors were intravenously injected with various formulations 4 times at 2-d intervals. Mice in the MB (+), MD NP (+), or MD1a NP (+) treatment group were subjected to NIR laser irradiation (660 nm, 500 mW/cm^2^, 2 min) 4 h postinjection. The equivalent doses of MB–DOX and 1a were 10 and 4.3 mg/kg, respectively. The volumes of both primary and distant tumors, along with body weight, were monitored every 2 d, with tumor volumes calculated as previously described. The DC maturation and T-cell activation in distant tumor tissues were analyzed through flow cytometry. The infiltration of CTLs and Tregs into distant tumor tissues was assessed by staining with CD8 and Foxp3, respectively.

### Statistical analysis

All quantitative data are presented as mean ± standard deviation. Data analysis was performed using the SPSS 25.0 software. Differences between groups were tested by one-way analysis of variance, and significant comparisons were conducted using Tukey’s multiple comparison test. The significance levels are indicated as follows: *P < 0.05, **P < 0.01, and ***P < 0.001, with “ns” indicating no significant difference.

## Results

### Preparation and characterization of MD1a NP

To synthesize MB–DOX, FDOCl-2 was first prepared and characterized by ^1^H nuclear magnetic resonance (NMR) according to reported methods (Figs. S1 and S2) [34]. Subsequently, MB–DOX was obtained via direct amidation of DOX with FDOCl-2. The chemical structure of MB–DOX was confirmed by high-resolution mass spectrometry, ^1^H NMR, and ^13^C NMR (Figs. S3 to S5). To evaluate HOCl responsiveness, the UV–vis absorption and fluorescence spectra of MB–DOX were analyzed. Upon exposure to HOCl (5 to 40 μM) in PBS (pH 7.4), MB–DOX exhibited a 38-fold enhancement in absorbance at 668 nm (Fig. S6A and B) and a 55-fold increase in fluorescence intensity at 700 nm (Fig. S6C and D). The detection limit for HOCl was determined to be 1.44 μM. Notably, in the MB–DOX prodrug, the loss of the conjugated groups in the MB moiety results in fluorescence quenching, whereas DOX fluorescence remains unaffected (Fig. S7). Moreover, MB–DOX demonstrated high selectivity for HOCl, as no significant increases in absorbance (Fig. S8A) or fluorescence (Fig. S8B) were observed in the presence of other ROS and RNS, including hydrogen peroxide (H_2_O_2_), peroxyl radical (O_2_^−^), singlet oxygen (^1^O_2_), hydroxyl radical (·OH), and peroxynitrite (ONOO^−^). Kinetic analysis reveals that the reaction between MB–DOX and HOCl (40 μM) occurs quickly, reaching completion within 75 s (Fig. S9).

**Fig. 2.**
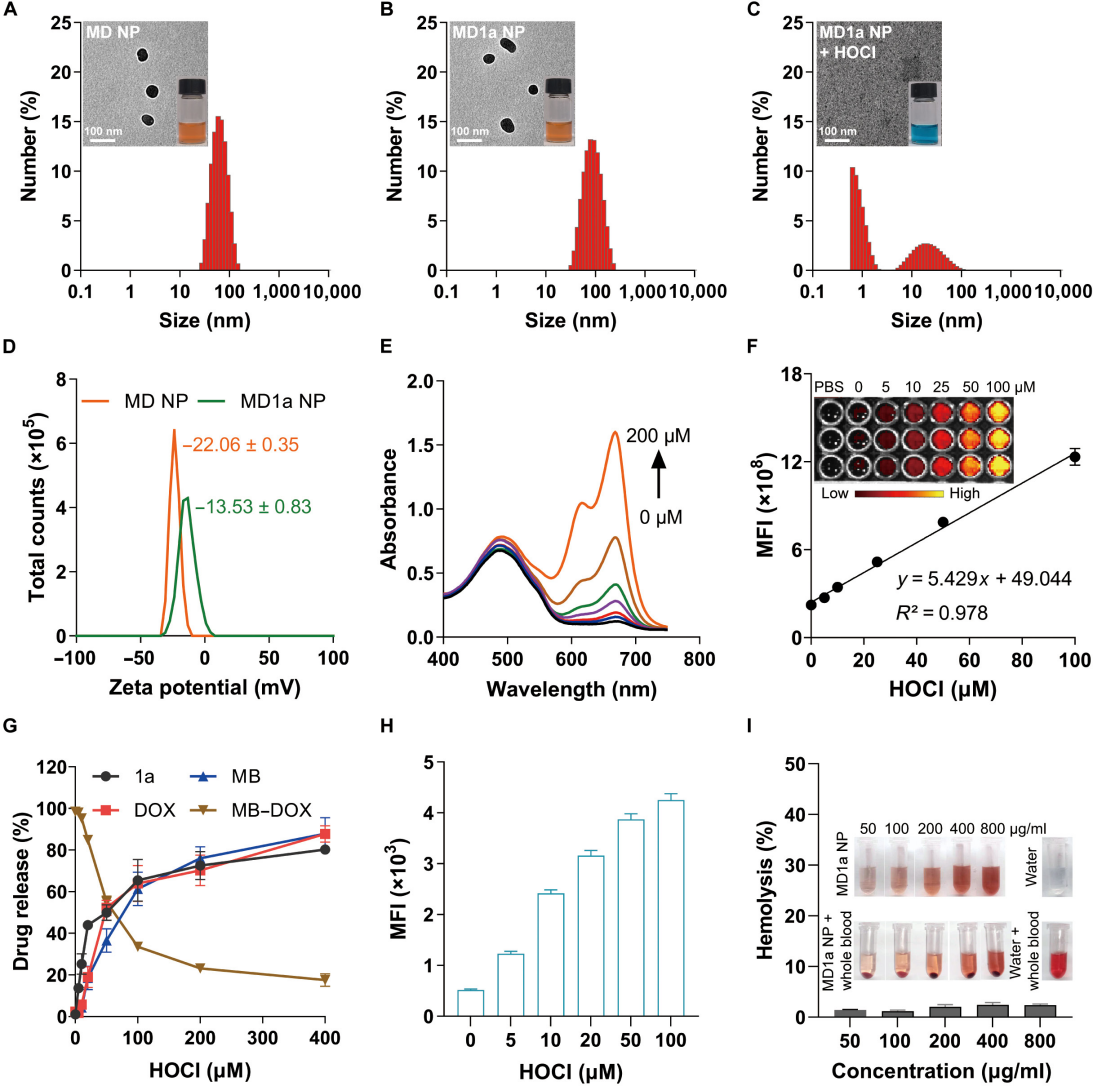
Characterization of MD1a NP. Representative transmission electron microscopy (TEM) images and hydrodynamic size distributions of (A) MD NP, (B) MD1a NP, and (C) MD1a NP with 10 μM HOCl treatment. (D) Zeta potential distributions of MD NP and MD1a NP. (E) Ultraviolet–visible (UV–vis) absorbance spectra of MD1a NP following HOCl (0 to 200 μM) treatment. (F) Linear correlation between the fluorescence intensity of MD1a NP at 700 nm and varying concentrations of HOCl (0 to 100 μM). Inset: Fluorescence image of MD1a NP solution treated with varying concentrations of HOCl. (G) Cumulative release profiles of MB, DOX, MB–DOX, and 1a from MD1a NP in response to varying concentrations of HOCl (0 to 400 μM). (H) Singlet oxygen generation by MD1a NP under laser irradiation after treatment with varying concentrations of HOCl (0 to 100 μM). (I) Hemolysis rates of MD1a NP at concentrations ranging from 50 to 800 μg/ml. Data are presented as mean ± SD (*n* = 3). MFI, mean fluorescence intensity; PBS, sodium phosphate buffer.

Subsequently, MD1a NP was fabricated by co-assembling MB–DOX and 1a via nanoprecipitation, with the incorporation of a phospholipid–polymer conjugate (DSPE-PEG_2000_) to enhance colloidal stability. The control nanoparticle (MD NP) was constructed similarly but without the inclusion of 1a. MD1a NP demonstrates high encapsulation efficiencies for MB–DOX and 1a, determined to be 83% and 67.4%, respectively. The drug-loading capacities were calculated as 40.8% for MB–DOX and 17.5% for 1a. Transmission electron microscopy images confirmed that both MD NP and MD1a NP exhibited monodisperse, uniform spherical morphologies (Fig. 2A and B). DLS analysis revealed that MD NP exhibited a hydrodynamic diameter of 67.2 nm (Fig. 2A). In comparison, the size of MD1a NP increased to 88.1 nm, confirming the successful encapsulation of 1a (Fig. 2B). Transmission electron microscopy and DLS analyses showed that MD1a NP disassembled into smaller particles upon treatment with 10 μM HOCl (Fig. 2C). The zeta potential of MD NP was determined to be −22.1 mV and increased to −13.5 mV upon incorporation of 1a (Fig. 2D). Both MD NP and MD1a NP exhibited a narrow size distribution, with polydispersity index (PDI) values determined to be 0.17 and 0.16, respectively. Notably, self-assembled nanoparticles formed by DSPE-PEG_2000_ alone (D NP) or by 1a and DSPE-PEG_2000_ (1a NP) exhibited average particle sizes of ~250 to 350 nm with relatively large PDIs (0.7 to 1.0), which were significantly higher than those of MD1a NP (Fig. S10). These results indicate that π–π stacking interactions between 1a and MB–DOX facilitate the formation of nanoparticles with smaller particle sizes and improved uniformity. MD NP and MD1a NP demonstrated good colloidal stability in PBS supplemented with 10% FBS, as demonstrated by negligible changes in hydrodynamic size (Fig. S11A) and PDI (Fig. S11B) over 1 week. Similarly, MD1a NP showed marked increases in UV–vis absorbance at 668 nm (Fig. 2E) and fluorescence intensity at 700 nm (Fig. 2F) in response to increasing concentrations of HOCl. HPLC was utilized to analyze the release of MB, DOX, and 1a from MD1a NP in PBS, with or without HOCl treatment. Approximately 80% of MB and DOX was released from MD1a NP upon exposure to 400 μM HOCl (Fig. 2G). Notably, HOCl stimulation significantly accelerated the release of 1a from MD1a NPs, confirming that HOCl-triggered nanoparticle disassembly facilitates 1a liberation (Fig. 2G and Fig. S12). Specifically, under 100 μM HOCl stimulation, an apparent release plateau was observed after approximately 60% release of 1a. This phenomenon is likely due to partial depletion of HOCl during the monitoring process, as it was simultaneously consumed through reaction with MB–DOX and underwent spontaneous decomposition, ultimately resulting in an insufficient concentration to further trigger drug release. In contrast, negligible MB–DOX activation and drug release were observed in the absence of HOCl over 24 h at 37 °C, confirming the ability to prevent premature release during systemic circulation (Fig. S13). The generation of singlet oxygen by MD1a NP was evaluated using SOSG as a fluorescence probe. As expected, a significant increase in SOSG fluorescence was observed in the MD1a NP solution upon NIR laser irradiation, with the fluorescence intensity dependent on both the HOCl concentration (Fig. 2H) and the duration of light exposure (Fig. S14). The hemolysis rate in MD1a NP-treated samples remained below 5%, even at the high concentration of 800 μg/ml, indicating excellent biocompatibility (Fig. 2I).

### Selective imaging and cytotoxicity of MD1a NP in cancer cells

**Fig. 3.**
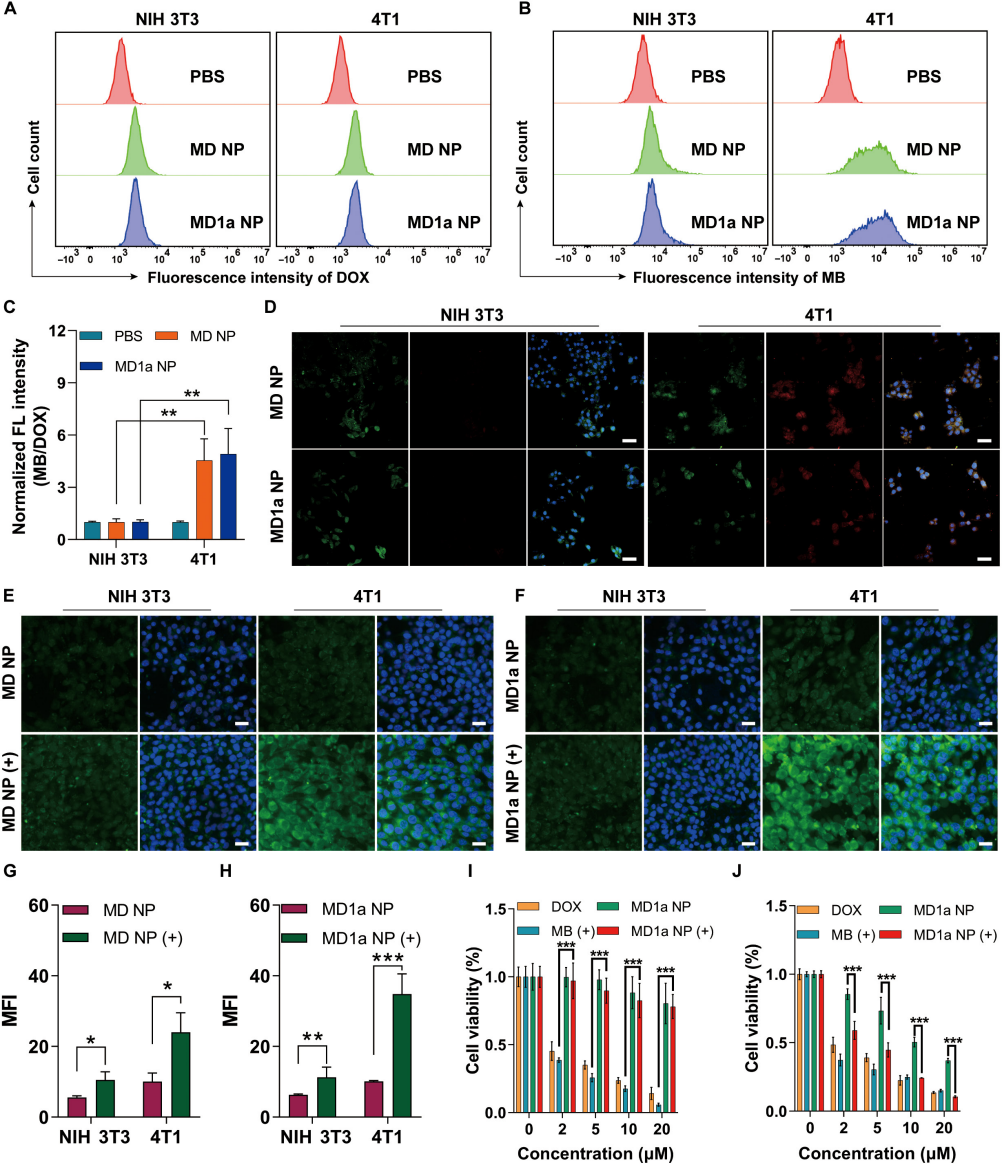
In vitro cytotoxicity of MD1a NP toward 4T1 cells. Representative flow cytometric histograms showing (A) DOX and (B) MB fluorescence signals in NIH 3T3 and 4T1 cells following different treatments. (C) Statistical analysis of the normalized MB-to-DOX fluorescence intensity ratio in NIH 3T3 or 4T1 cells (*n* = 3). (D) Representative confocal laser scanning microscopy (CLSM) images showing MB and DOX fluorescence signals in NIH 3T3 and 4T1 cells following different treatments. Blue, 4′,6-diamidino-2-phenylindole (DAPI); green, DOX fluorescence; red, MB fluorescence. Scale bar = 50 μm. Representative CLSM images showing intracellular singlet oxygen levels in 4T1 and NIH 3T3 cells treated with (E) MD NP and (F) MD1a NP, with or without NIR laser irradiation. Blue, DAPI; green, Singlet Oxygen Sensor Green (SOSG) fluorescence. Scale bar = 25 μm. Quantification of intracellular singlet oxygen levels (G) in panel (E) and (H) in panel (F) (*n* = 3). Cell viability of (I) NIH 3T3 cells and (J) 4T1 cells following different treatments. Data are represented as mean ± SD. ns, no significance; *P < 0.05; **P < 0.01; ***P < 0.001. FL, fluorescence.

**Fig. 4.**
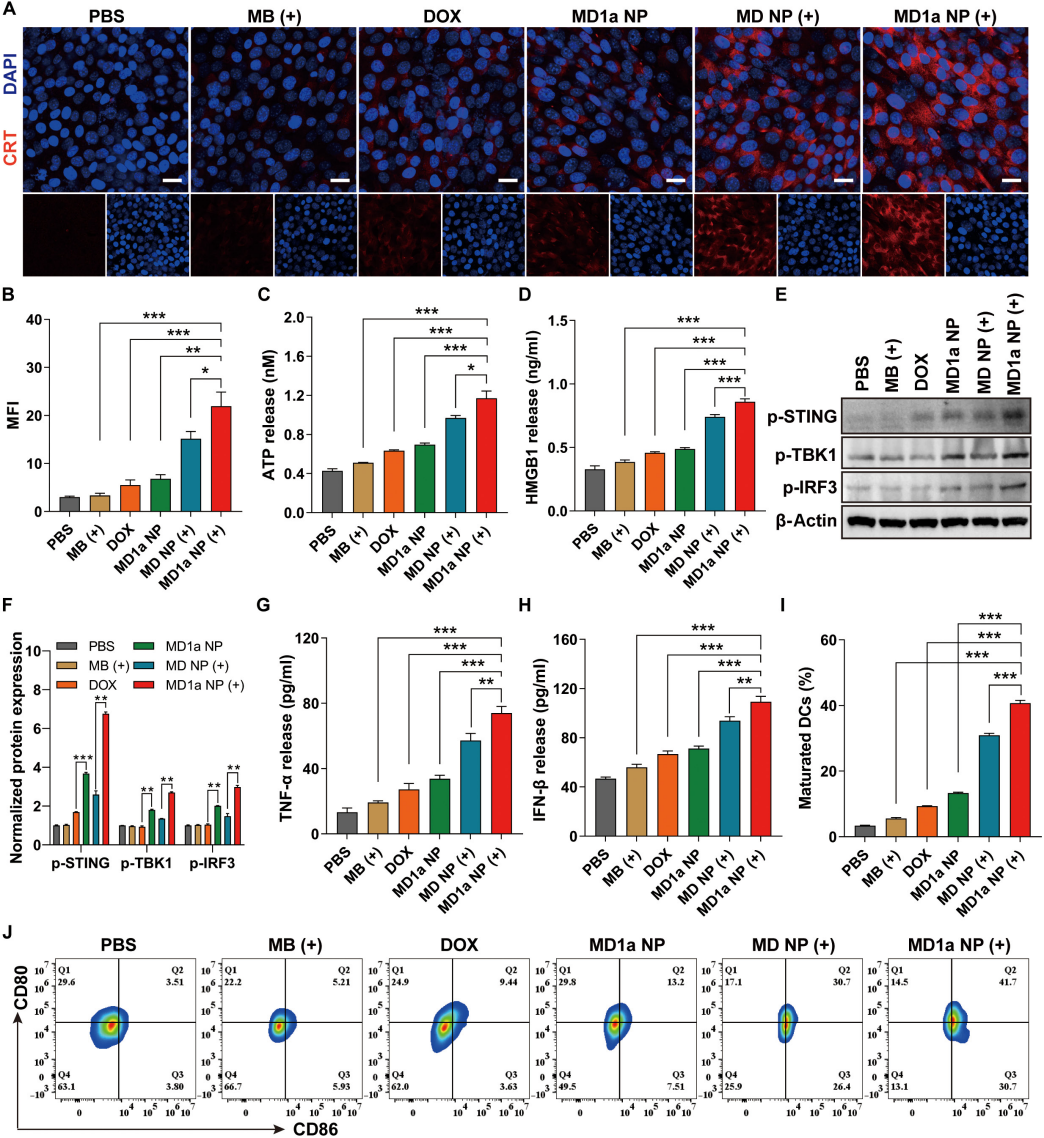
MD1a NP-induced ICD and STING activation in 4T1 cells. (A) Representative CLSM images showing calreticulin (CRT) exposure on 4T1 cell membranes across different groups. Scale bar = 25 μm. (B) Quantification of CRT fluorescence intensity in panel (A). Release of (C) adenosine triphosphate (ATP) and (D) HMGB1 by 4T1 cells following different treatments. (E) Western blot analysis of STING-pathway-associated proteins in 4T1 cells following different treatments. (F) Normalized expression levels of p-STING, p-TBK1, and p-IRF3 in 4T1 cells following different treatments. Secretion of (G) tumor necrosis factor-α (TNF-α) and (H) interferon-beta (IFN-β) by 4T1 cells in a transwell co-culture system under different treatment conditions. (I) Averaged frequency and (J) representative flow cytometry plots of matured DCs across different groups. Data are represented as mean ± SD (*n* = 3). *P < 0.05; **P < 0.01; ***P < 0.001.

To study the intracellular performance of MD1a NP, the normal embryonic mouse fibroblast cell line NIH 3T3 was used as a control. Flow cytometry analysis revealed that the MB-to-DOX fluorescence intensity ratio in 4T1 cells was higher than that in NIH 3T3 cells (Fig. 3A to C). CLSM images further confirmed that both 4T1 and NIH 3T3 cells exhibited comparable DOX fluorescence following treatment with MD NP or MD1a NP (Fig. 3D). This is due to the retention of DOX fluorescence in the MB–DOX prodrug. However, the MB fluorescence was significantly stronger in 4T1 cells than in NIH 3T3 cells, indicating greater activation of MB in cancer cells. This result is consistent with previous reports describing a HOCl-responsive probe (MB–PEG–Bio2) that possesses the same HOCl-responsive moiety as MB–DOX and is specifically activated in 4T1 cells [31]. The ability of MD1a NP to generate ROS in 4T1 and NIH 3T3 cells was assessed using DCFH-DA as a probe (Fig. S15). CLSM images showed that both MD NP and MD1a NP significantly enhanced the production of ROS in 4T1 cells upon NIR laser irradiation (+), as indicated by the increased fluorescence of DCFH-DA. In contrast, NIH 3T3 cells treated with MD NP (+) or MD1a NP (+) displayed weak DCFH-DA fluorescence. Notably, the DCFH-DA probe is not a specific probe for singlet oxygen; we then used the fluorescent probe SOSG to specifically monitor intracellular singlet oxygen. Representative CLSM images showed that both MD NP (+) and MD1a NP (+) significantly increased singlet oxygen levels in 4T1 cells compared with the control group (Fig. 3E to H). The cytotoxic effects of MD1a NP on NIH 3T3 (Fig. 3I) and 4T1 cells (Fig. 3J) were evaluated using the standard CCK-8 assay. MD1a NP and MD1a NP (+) treatments exhibited minimal cytotoxicity toward NIH 3T3 cells across various MB–DOX concentrations. In contrast, treatment with free DOX and MB (+) at the equivalent concentrations resulted in obvious cytotoxicity in NIH 3T3 cells. The viability of 4T1 cells decreased to below 40% and 10% following treatment with MD1a NP and MD1a NP (+), respectively. Notably, D NP showed no detectable cytotoxicity even at 20 μg/ml (Fig. S6A). Similarly, free 1a exhibited no significant inhibitory effect on 4T1 cell proliferation within the 1 to 20 μM concentration range (Fig. S16B). These results demonstrate the potential of MD1a NP for tumor-specific imaging and tumor-activatable photochemotherapy.

### ICD induction and DC maturation

We next investigated the potential of MD1a NP to induce ICD in 4T1 cells by assessing ICD-associated markers. Specifically, the translocation of CRT from the endoplasmic reticulum to the cell surface was evaluated via immunofluorescence staining (Fig. 4A and B). Compared to the PBS group, stronger CRT fluorescence was observed in the MD NP (+) and MD1a NP (+) groups. Additionally, the extracellular release of HMGB1 and ATP from 4T1 cells under various treatments was measured using ELISA and ATP assay kits, respectively. Both MD NP (+) and MD1a NP (+) treatments significantly enhanced the secretion of ATP (Fig. 4C) and HMGB1 (Fig. 4D) compared to the MB (+) and DOX groups, indicating that the synergistic photochemotherapy mediated by MD1a NP was more effective at inducing ICD than monotherapy. Western blot analysis revealed that the normalized expression levels of phosphorylated STING (p-STING), TBK1 (p-TBK1), and IRF3 (p-IRF3) were significantly upregulated in the MD1a NP group compared to those in the other groups (Fig. 4E and F). Next, we established a transwell co-culture system to determine whether MD1a NP (+)-induced ICD, in combination with 1a-mediated STING activation, could enhance DC maturation. Briefly, 4T1 cells with various treatments were seeded into the upper chamber and co-cultured with BMDCs in the lower chamber. Tumor antigens, DAMPs, DNA fragments, and 1a released from the treated 4T1 cells were able to diffuse into the lower chamber and be internalized by DCs. After 36 h of co-incubation, the levels of pro-inflammatory cytokines, including TNF-α and IFN-β, in the co-culture system were quantified using ELISA kits. Compared to the PBS group, 4T1 cells treated with MD1a NP (+) exhibited significant increases in TNF-α and IFN-β secretion by 5.7-fold (Fig. 4G) and 2.4-fold (Fig. 4H), respectively. Flow cytometry was used to evaluate DC maturation by measuring the expression of key surface markers CD80 and CD86 (Fig. 4I and J). Compared to the PBS group, treatment with free MB (+) or DOX led to 1.7-fold and 2.8-fold increases in DC maturation, respectively. Notably, the proportion of mature DCs in the MD1a NP group was approximately 4-fold higher than that in the PBS group and was further increased to a 12-fold enhancement upon NIR light irradiation.

### In vivo biodistribution

**Fig. 5.**
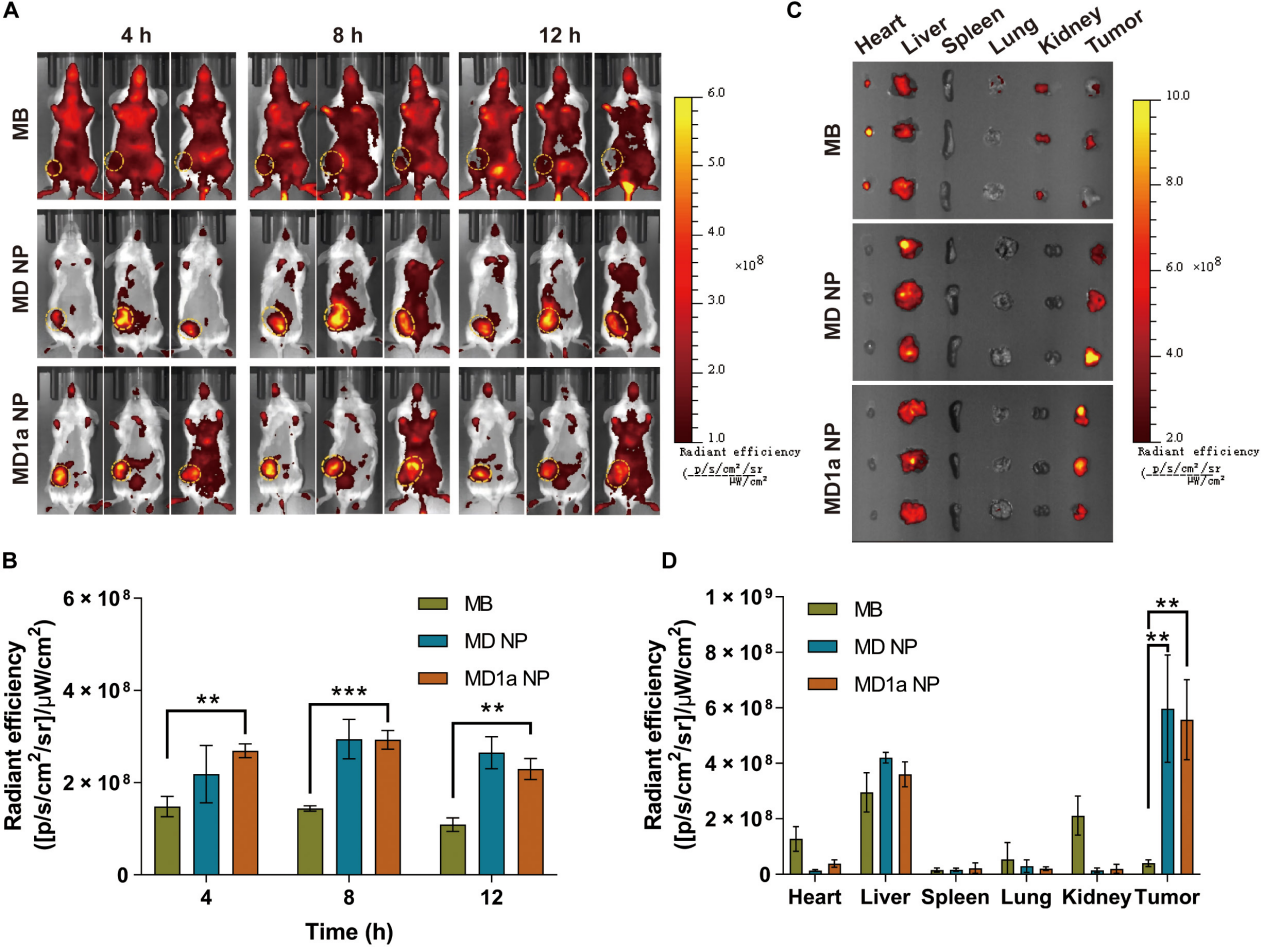
In vivo activation and biodistribution of MD1a NP in an orthotopic 4T1 breast cancer model. (A) Fluorescence images of 4T1 tumor-bearing mice following different treatments. (B) Quantification of tumor-localized MB fluorescence intensity at different time points in panel (A). (C) Fluorescence images of tumors and major organs across different groups. (D) Semiquantitative analysis of MB fluorescence intensity in tumors and major organs. Data are represented as mean ± SD (*n* = 3). **P < 0.01; ***P < 0.001.

To investigate tumor-specific activation, MD NP and MD1a NP were intravenously injected into an orthotopic 4T1 tumor-bearing mouse model at an equivalent MB dose of 1.4 mg/kg. Free MB was used as a control to evaluate the tumor accumulation efficiency of the free drug. NIR fluorescence imaging was conducted at different time points (4, 8, and 12 h) postinjection using the IVIS imaging system (Fig. 5A). In the free MB treatment group, fluorescence signals were distributed throughout the body, with the pronounced fluorescence intensity observed in the bladder region, likely due to the rapid renal clearance of the small-molecule MB. Moreover, IVIS imaging demonstrates that free MB undergoes slow elimination following intravenous administration, supporting the rationale for our design of the MB–DOX prodrug. In contrast, the MD1a NP group exhibited higher tumor-localized MB fluorescence than the free MB group at different time points postinjection (Fig. 5B). To further evaluate the in vivo activation of MD1a NP and restoration of MB fluorescence, tumors and major organs were harvested for ex vivo imaging at 12 h postinjection. In the free MB group, obvious MB fluorescence signals were detected in the heart, liver, kidney, and tumors (Fig. 5C and D). By comparison, the MD1a NP group exhibited significantly higher tumor-localized MB fluorescence compared to other major organs, demonstrating enhanced tumor specificity. Representative CLSM images demonstrated that the MB-to-DOX fluorescence intensity ratio in tumor tissues treated with MD1a NP was higher than that in the liver (Fig. S17). To further elucidate the in vivo drug release mechanism of MD NP and MD1a NP, we compared HOCl levels in tumor tissues with those in normal organs using the HOCl-specific fluorescent probe EtS-DMAB (Fig. S18). These results revealed higher HOCl levels in tumors compared with those in normal tissues, including the heart, liver, spleen, lung, and kidney.

### In vivo anticancer activity

**Fig. 6.**
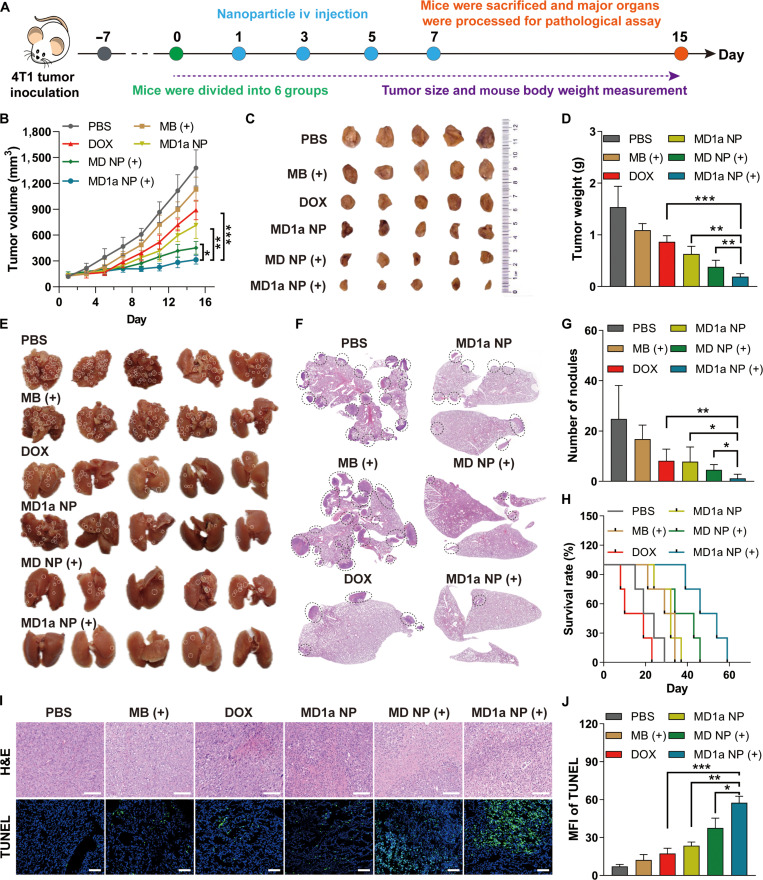
In vivo antitumor effects of MD1a NP. (A) Illustration of the in vivo treatment procedure. (B) Tumor growth curves, (C) tumor photographs, and (D) average tumor weights across different groups (*n* = 5). (E) Lung images, (F) representative hematoxylin and eosin (H&E) staining of lung tissues and (G) quantification of pulmonary metastatic nodules across different groups (*n* = 5). (H) Survival curves of 4T1 tumor-bearing mice following different treatments (*n* = 8). (I) Representative H&E and terminal deoxynucleotidyl transferase-mediated dUTP nick-end labeling (TUNEL) staining of tumor tissues across different groups (*n* = 3). Scale bar = 50 μm. Blue, DAPI; green, TUNEL. (J) Quantification of TUNEL fluorescence intensity in panel (I). Data are represented as mean ± SD. *P < 0.05; **P < 0.01; ***P < 0.001. iv, intravenous.

The in vivo antitumor efficacy of MD1a NP was evaluated using an orthotopic 4T1 breast tumor model, with the treatment regimen illustrated in Fig. 6A. When tumors reached approximately 150 mm^3^, mice were randomly assigned to different groups and intravenously injected with PBS, free MB, free DOX, MD NP, or MD1a NP. The equivalent doses of MB–DOX and 1a were 10 and 4.3 mg/kg, respectively. For groups receiving light treatment (+), tumors were irradiated with an NIR laser 4 h postinjection. The mice were treated with different formulations once every 2 d, based on the plasma half-lives of DOX and MB (Fig. S19). Treatment with MB (+), DOX, and MD NP (+) resulted in tumor growth inhibition rates of approximately 18%, 35%, and 67%, respectively (Fig. 6B and C). Notably, MD1a NP treatment achieved a tumor inhibition rate of 48%, which further increased to approximately 77% upon NIR laser irradiation. The enhanced antitumor effect was likely attributed to the synergistic induction of ICD facilitated by the combined PDT and chemotherapy. In addition, MD1a NP (+) treatment resulted in an approximately 88% reduction in tumor weight compared to the PBS group (Fig. 6D). No significant body weight loss (Fig. S20) or pathological abnormalities (Fig. S21) were observed in major organs (heart, liver, spleen, lungs, and kidneys) in either the MD NP or the MD1a NP group, indicating good biocompatibility. Serum biochemical analysis demonstrated that mice treated with MD NP or MD1a NP exhibited significantly lower systemic toxicity compared with those receiving free DOX (Fig. S22). We next evaluated the ability of MD1a NP (+) treatment to inhibit tumor metastasis (Fig. S23). Images and H&E staining of lung tissues showed a significant reduction in pulmonary metastatic foci in the MD1a NP (+) group compared to those in the PBS group (Fig. 6E to G). These findings suggest that MD1a NP (+) treatment may elicit systemic antitumor immune responses that effectively inhibit lung metastasis. Survival analysis showed that mice treated with DOX exhibited shorter survival times than those in the PBS group, likely due to systemic cytotoxicity associated with free DOX (Fig. 6H). Although mice in the MB (+) and MD NP treatment groups revealed modest improvements in survival, the persistent decline in survival rates underscores the limitations of single-treatment approaches and the systemic off-target effects of free drugs. In contrast, MD1a NP (+) treatment significantly prolonged the survival of 4T1 tumor-bearing mice compared to other groups. H&E staining of tumor tissues revealed increased apoptosis in the MD1a NP (+) group, characterized by pronounced nuclear condensation and cytoplasmic loss (Fig. 6I). Additional analyses using TUNEL immunostaining further confirmed a high proportion of apoptotic cells and a marked reduction in proliferating tumor cells in the MD1a NP (+) group (Fig. 6I and J).

### Remodeling of the tumor immune microenvironment

**Fig. 7.**
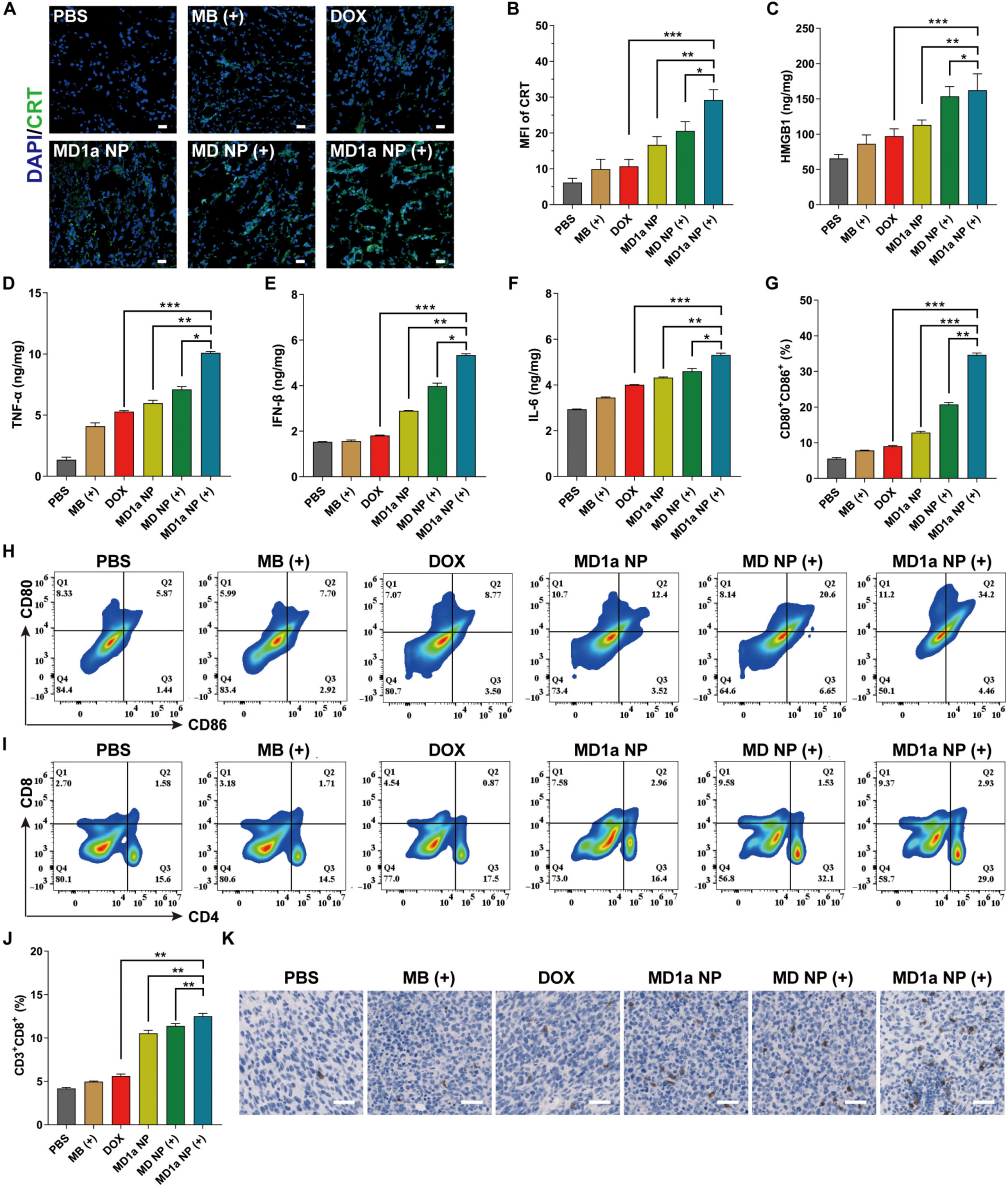
In vivo immune activation induced by MD1a NP. (A) Representative immunofluorescence images and (B) quantification of CRT expression in tumor tissues across different groups. Scale bars = 20 μm. The release of (C) HMGB1, (D) TNF-α, (E) IFN-β, and (F) interleukin-6 (IL-6) in tumor tissues following different treatments. (G) Frequency and (H) representative flow cytometry plots of mature DC (CD80^+^CD86^+^) in tumor tissues following different treatments. (I) Representative flow cytometry plots and (J) quantification of CD3^+^CD8^+^ T cells in tumor tissues following different treatments. (K) Representative immunohistochemical (IHC) images of CD8^+^ T-cell infiltration in tumor tissues. Scale bar = 25 μm. Data are presented as mean ± SD (*n* = 3). ns, no significance; *P < 0.05; **P < 0.01; ***P < 0.001.

We next evaluated the in vivo immunostimulatory activity of MD1a NP. Representative CLSM images (Fig. 7A and B) and flow cytometry analysis (Fig. S24) demonstrated that MD1a NP (+) treatment markedly enhanced CRT exposure in tumor tissues. Moreover, intratumoral HMGB1 release was significantly elevated in both the MD NP (+) and MD1a NP (+) groups compared with those in other treatments (Fig. 7C). Collectively, these findings indicate that synergistic PDT and chemotherapy mediated by MD1a NP (+) induce a more potent ICD response than monotherapies. Pro-inflammatory cytokines such as TNF-α, IFN-β, and IL-6 were significantly elevated in tumor tissues (Fig. 7D to F) and serum (Fig. S25) compared to those in the PBS group. Flow cytometry analysis revealed that the MD1a NP (+) group exhibited the highest level of DC maturation (34.2%), which was higher than the 20.6% and 12.4% observed in the MD NP (+) and MD1a NP groups, respectively (Fig. 7G and H). Increased production of pro-inflammatory cytokines within tumors has been shown to promote the recruitment of immune cells, particularly CTLs, which are critical for eliminating cancer cells. Moreover, tumor infiltration of CD8^+^ T cells was significantly enhanced following MD1a NP (+) treatment, showing 1.86-fold and 1.38-fold increases compared to those of the DOX and MD NP (+) groups, respectively (Fig. 7I and J). Compared with the other groups, MD1a NP treatment significantly decreased the proportion of Tregs at the tumor site (Fig. S26A and C) and markedly promoted memory T-cell formation in mice (Fig. S26B and D). IHC analysis further confirmed the increased infiltration of CD8^+^ T cells in tumors after MD1a NP (+) treatment (Fig. 7K). These results suggest that MD1a NP, followed by NIR laser irradiation, elicits potent antitumor immunity.

### Immune abscopal effects induced by MD1a NP

**Fig. 8.**
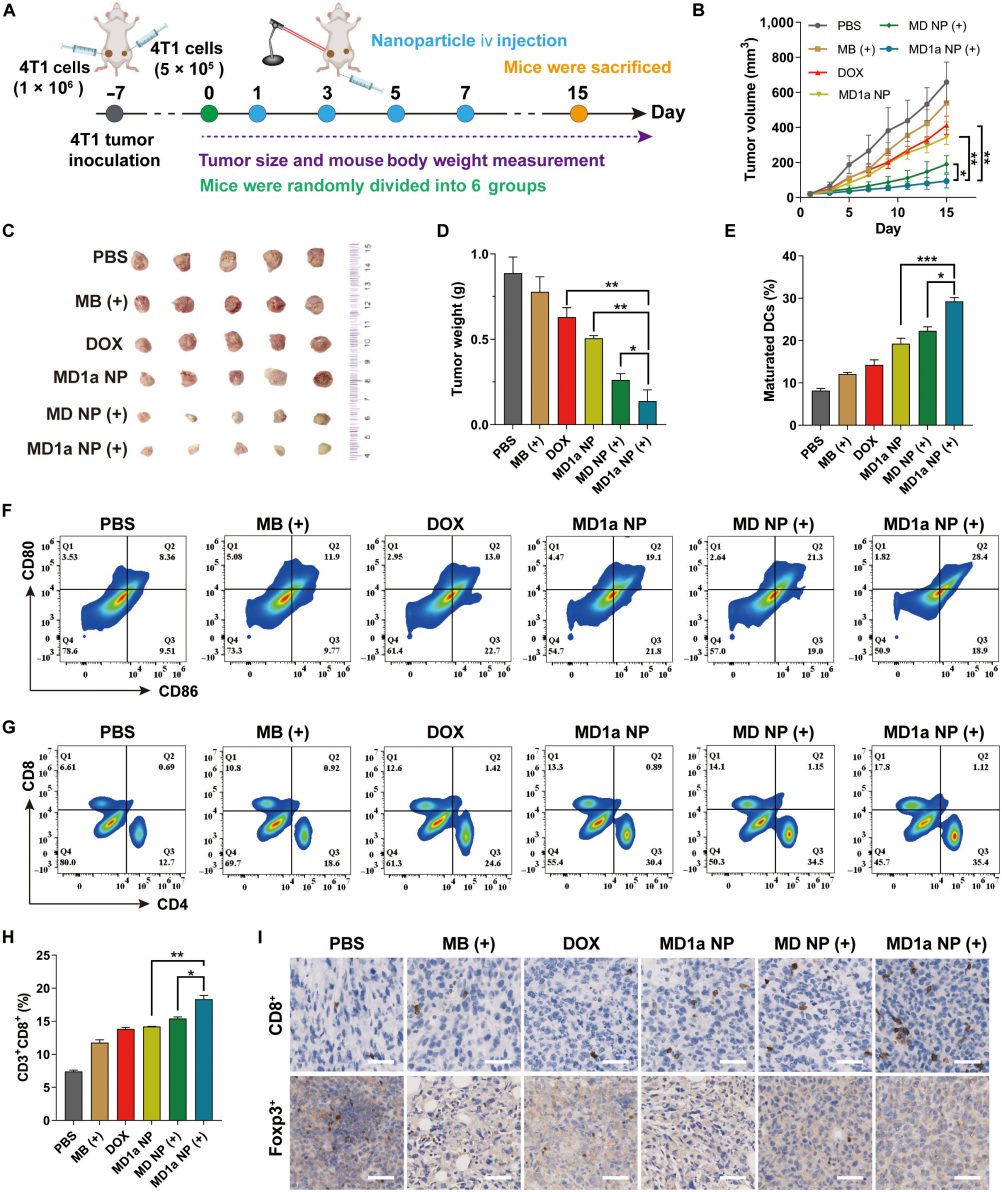
Antitumor effects of MD1a NP in a syngeneic bilateral 4T1 breast cancer model. (A) Schematic illustration of the treatment schedule. (B) Growth curves, (C) photographs, and (D) average weights of distant tumors across different groups (*n* = 5). (E) The proportions and (F) representative flow cytometry plots of mature DC (CD80^+^CD86^+^) within distant tumors following different treatments (*n* = 3). (G) Representative flow cytometry plots showing the proportions of T cells (CD3^+^CD8^+^CD4^+^) within distant tumors following different treatments (*n* = 3). (H) Quantification of the proportions of CD3^+^CD8^+^ T cells in tumor tissues (*n* = 3). (I) Representative immunofluorescence staining of CD8^+^ T cells and Foxp3^+^ Tregs in distant tumors following various treatments (*n* = 3). Scale bars = 25 μm. All data are presented as mean ± SD. *P < 0.05; **P < 0.01; ***P < 0.001.

The abscopal antitumor effects of MD1a NP were further investigated using a bilateral orthotopic 4T1 tumor model (Fig. 8A). DOX and MB (+) groups showed minimal effects on distant tumor growth, with tumor burdens comparable to those observed in the PBS group (Fig. 8B to D). In contrast, MD1a NP (+) treatment not only reduced primary tumor burden (Fig. S27) but also effectively inhibited the growth of distant tumors. To elucidate the mechanism underlying MD1a NP-mediated inhibition of distant tumor growth, we performed immunophenotypic analysis of distant tumor tissues by flow cytometry. The MD1a NP (+) group exhibited a nearly 1.5-fold increase in mature DCs in distant tumor tissues compared to the MD1a NP group, underscoring the pivotal role of PDT in enhancing antitumor immune responses (Fig. 8E and F). Flow cytometric (Fig. 8G and H) and IHC analysis (Fig. 8I) further demonstrated significantly increased infiltration of CD8^+^ T cells into distant tumors in the MD1a NP (+) group compared to those in the other treatment groups. MD1a NP (+) treatment significantly reduced the infiltration of Tregs in distant tumors, indicating an alleviation of the immunosuppressive TME (Fig. 8I). Collectively, these results indicate that MD1a NP combined with NIR laser irradiation induced potent systemic and distant immune responses, leading to a substantial reduction in distant tumor burden.

## Conclusion

In summary, we developed a multifunctional nanoassembly (MD1a NP) by co-assembling a HOCl-responsive dimer prodrug, MB–DOX, with a STING agonist, 1a, to elicit personalized antitumor immunity and overcome tumor immunosuppression. In response to elevated HOCl levels in tumor tissues, MB and DOX are activated and released from MD1a NP, enabling tumor-activatable photochemotherapy under NIR laser irradiation. This combinatorial therapy induces robust ICD, thereby promoting tumor antigen exposure. Moreover, HOCl-triggered disassembly of MD1a NP facilitates the release of 1a, which activates the STING pathway and remodels the TME toward an immune-supportive state. In orthotopic 4T1 breast cancer models, MD1a NP treatment effectively reduced the proportion of Tregs, alleviated T-cell immunosuppression, and promoted the formation of memory T cells. Consequently, MD1a NP combined with NIR laser irradiation reversed the immunosuppressive TME and elicited systemic antitumor immunity, thereby inhibiting both primary and abscopal tumor growth, preventing lung metastasis, and prolonging overall survival. This study provides a promising strategy for developing tumor-responsive nanomedicines to achieve synergistic immunotherapy against immune-desert cancers.

## Data Availability

The data that support the findings of this study are available on request from the corresponding authors.
