# Supplementary material for: Hypochlorous Acid-Responsive Prodrug Nanoplatform for Synergistic Cancer Immunotherapy
Source: Biomater Res. 2026 Jan 23;30:0300. doi: 10.34133/bmr.0300 (PMC12827883; doi:10.34133/bmr.0300)
Supplement: Supplementary 1 — Figs. S1 to S27 [file bmr.0300.f1.docx]

**Hypochlorous Acid-Responsive Prodrug Nanoplatform for** **Synergistic Cancer Immunotherapy**

*Shu Xia,^1†^ Xinyu Wang,^1†^ Cheng Liu,^1^ Ran Ji,^2^ Mingzhi Wang,^2,3^ Chi Zhang,^4^ Liang Chen,^2,3^ Wenqiang Chen,^*4^ Shao Q. Yao,^5^ Chao Fang^*6^and Xiao Dong^*1, 2, 3^*

1 Shanghai 411 Hospital, China RongTong Medical Healthcare Group Co. Ltd./411 Hospital, Shanghai University, Shanghai, 200081, China.

E-mail: [dong-xiao@shu.edu.cn](mailto:dong-xiao@shu.edu.cn)

2 Institute of Artificial Intelligence and Biomanufacturing, School of Medicine, Shanghai University, Shanghai 200444, China.

3 Shanghai Tenth People's Hospital of Tongji University, Shanghai 200072, China.

4 Guangxi Key Laboratory of Natural Polymer Chemistry and Physics, College of Chemistry and Materials Science, Nanning Normal University, Nanning, 530001, China.

E-mail: [chenwqAP@163.com](mailto:chenwqAP@163.com)

5 Department of Chemistry, National University of Singapore, 3 Science Drive 3, Singapore 117543, Singapore.

6 Hongqiao International Institute of Medicine, Tongren Hospital and State Key Laboratory of Systems Medicine for Cancer, Shanghai Jiao Tong University School of Medicine, Shanghai, 200025 China.

E-mail: [fangchao32@sjtu.edu.cn](mailto:fangchao32@sjtu.edu.cn)

† Shu Xia and Xinyu Wang contributed equally to this work.

**Supporting Information**


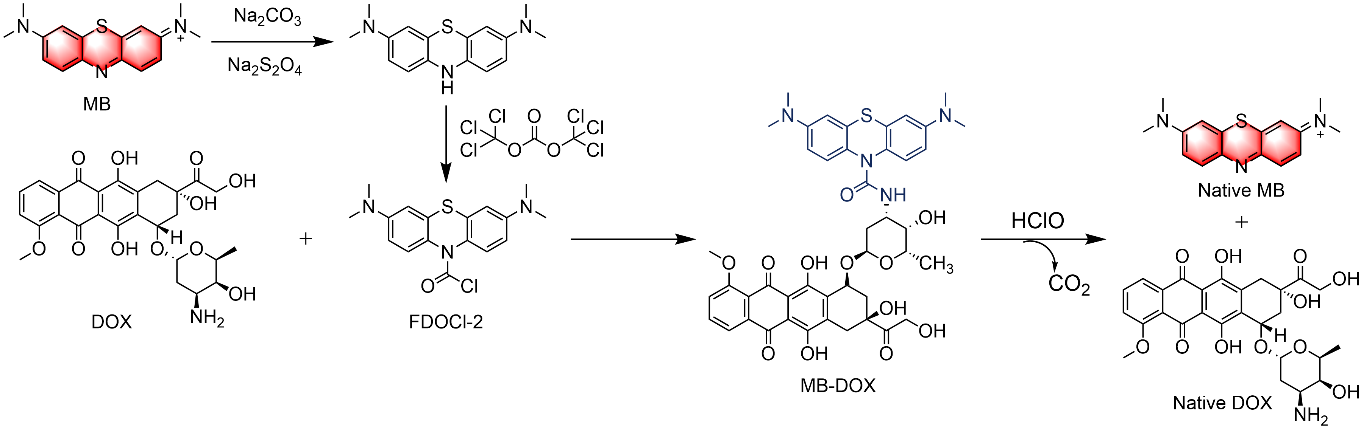


**Figure S1**. The synthesis routes of MB-DOX.


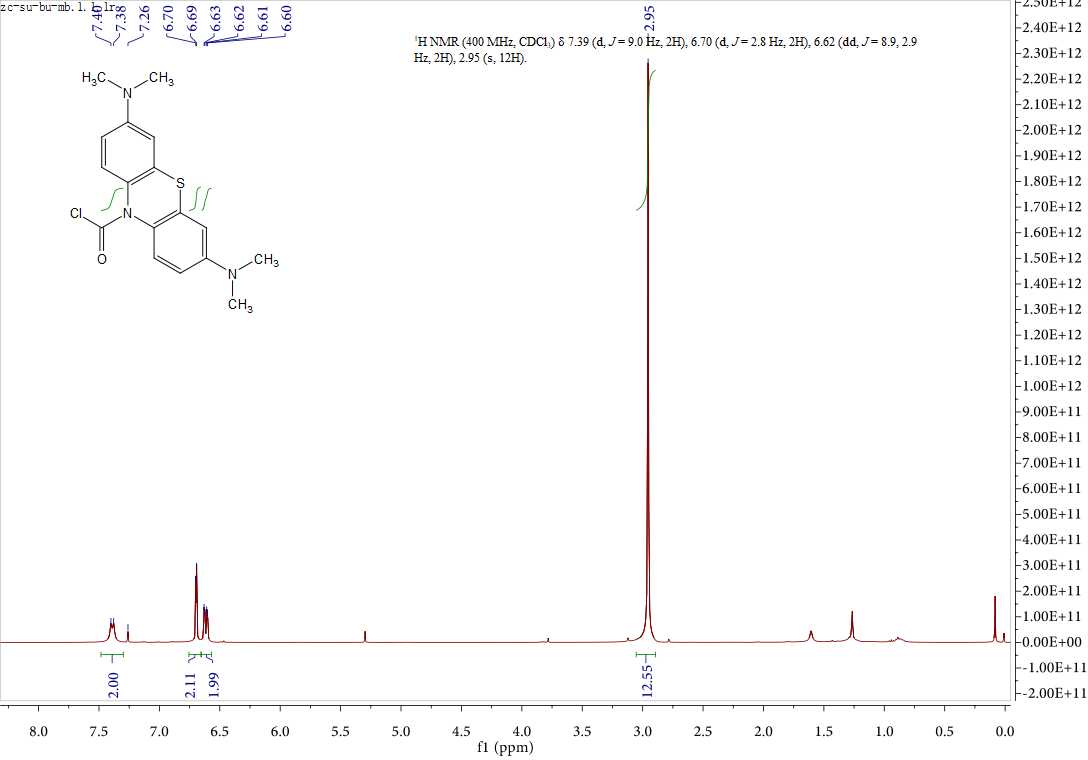


**Figure S2**. ^1^HNMR spectra of FDOCl-2. ^1^H NMR (400 MHz, CDCl_3_) δ 7.39 (d, *J* = 9.0 Hz, 2H), 6.70 (d, *J* = 2.8 Hz, 2H), 6.62 (dd, *J* = 8.9, 2.9 Hz, 2H), 2.95 (s, 12H).


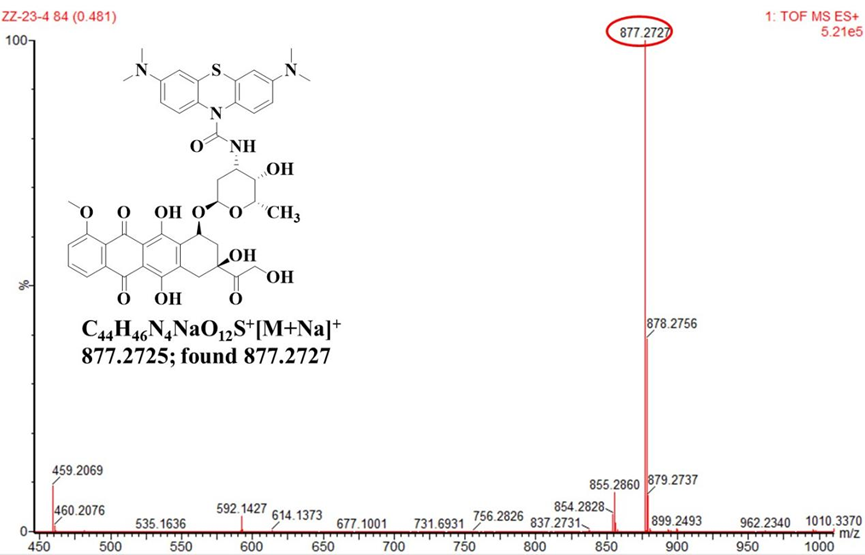


**Figure S3**. LCMS spectrum of MB-DOX. HRMS (ESI) m/z for C_44_H_46_N_4_NaO_12_S^+^ [M+Na]^+^ 877.2725; found 877.2727.

**Figure S4**. ^1^HNMR spectra of MB-DOX. Mp: ^1^H NMR (400 MHz, Chloroform-*d*) δ 13.91 (s, 1H), 13.19 (s, 1H), 8.00 (d, *J* = 7.6 Hz, 1H), 7.75 (t, *J* = 8.1 Hz, 1H), 7.36 (d, *J* = 8.4 Hz, 1H), 7.29 – 7.22 (m, 3H), 6.60 (d, *J* = 2.1 Hz, 2H), 6.57 – 6.50 (m, 2H), 5.49 – 5.43 (m, 1H), 5.30 (d, *J* = 8.0 Hz, 1H), 5.23 (s, 1H), 4.73 (s, 2H), 4.58 (s, 1H), 4.11 (q, *J* = 6.2 Hz, 1H), 4.05 (s, 4H), 3.71 (s, 1H), 3.21 (d, *J* = 18.8 Hz, 1H), 3.07 – 2.99 (m, 1H), 2.87 (s, 12H), 2.31 (d, *J* = 14.9 Hz, 1H), 2.21 – 2.07 (m, 2H), 1.90 – 1.64 (m, 5H).

**Figure S5**. ^13^CNMR spectra of MB-DOX. ^13^C NMR (101 MHz, CDCl_3_) δ 213.0, 185.9, 185.5, 159.9, 155.2, 154.6, 154.3, 147.8, 134.6, 134.4, 132.9, 132.7, 132.6, 127.0, 125.9, 119.8, 118.7, 117.4, 110.4, 110.2, 110.2, 109.8, 99.9, 75.4, 68.6, 68.5, 66.4, 64.5, 55.6, 46.0, 39.6, 34.7, 32.9, 30.9, 30.6, 29.2, 29.1, 28.6, 28.6, 28.3, 28.2, 21.6, 15.8, 13.1.


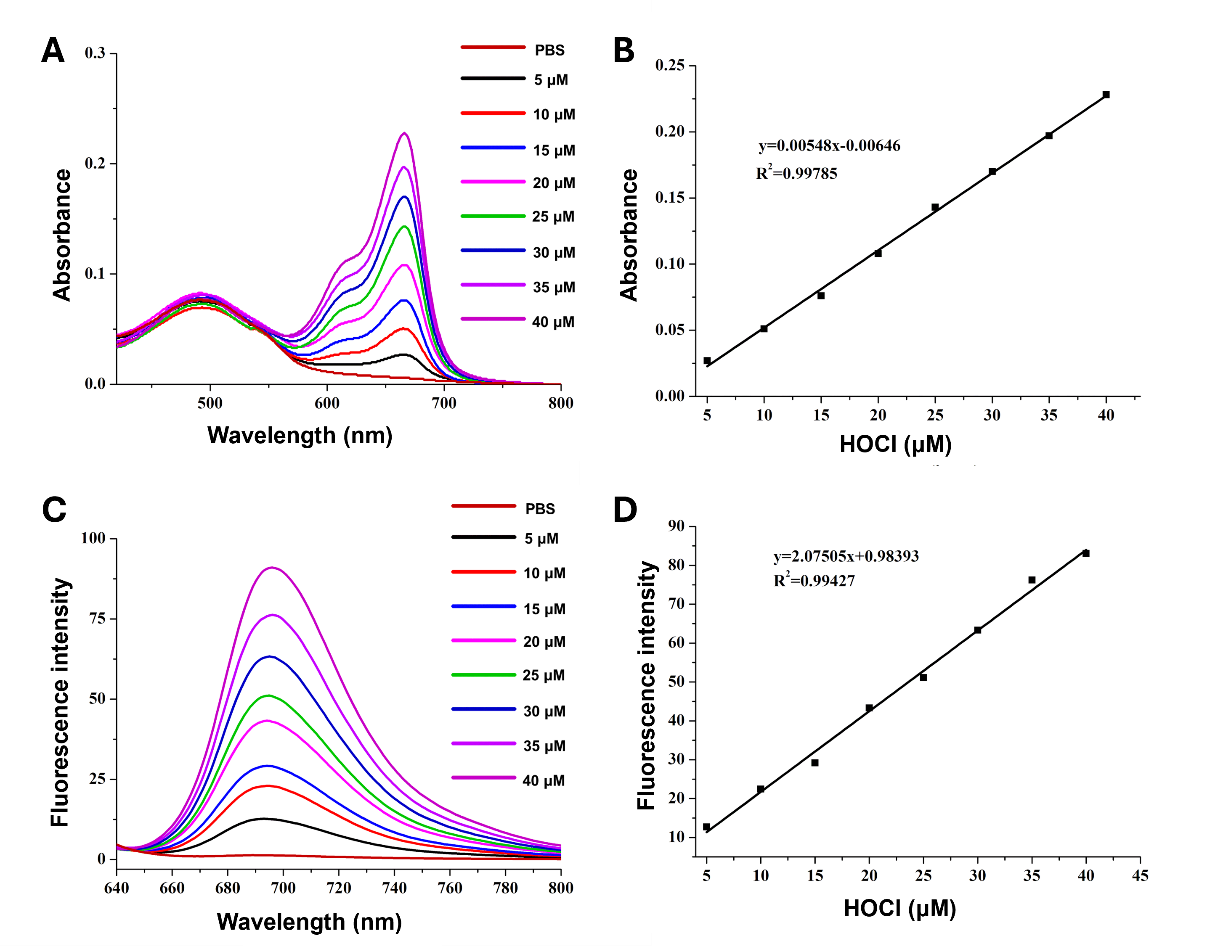


**Figure S6**. Characterization of MB-DOX in response to varying concentrations of HOCl. (**A**) Changes in the UV-vis absorption spectrum of MB-DOX with varying HOCl concentrations (5 μM to 40 μM). (**B**) Linear correlation between HOCl concentration and UV-vis absorbance of MB-DOX at 668 nm. (**C**) Fluorescence spectrum of MB-DOX after treatment with varying HOCl concentrations (5 μM to 40 μM). (**D**) Linear correlation between HOCl concentration and fluorescence intensity of MB-DOX at 700 nm.


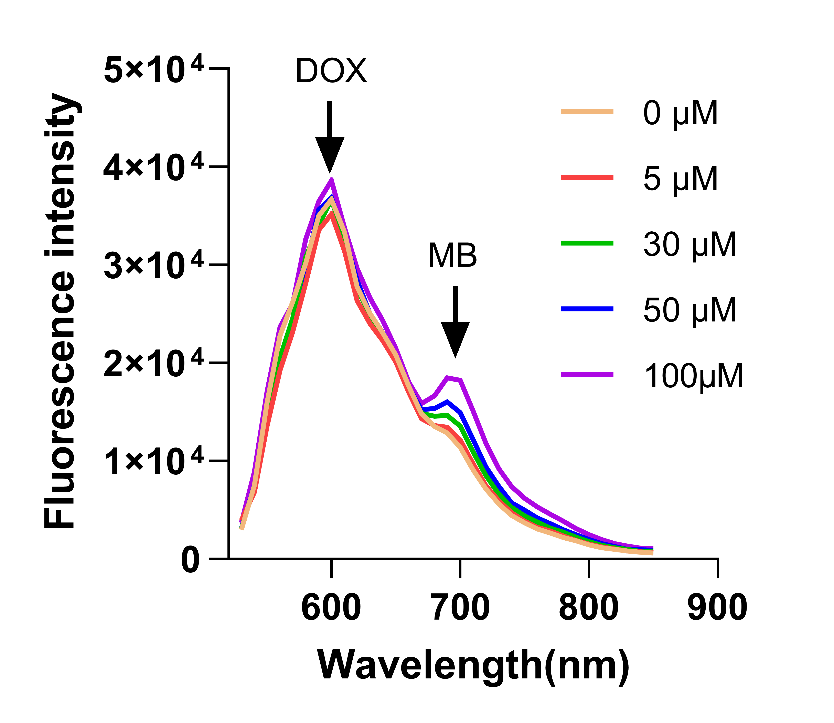


**Figure S7.** Fluorescence spectra of MB–DOX measured from 520 to 700 nm under excitation at 480 nm in the presence of varying concentrations of HOCl (5–100 μM).


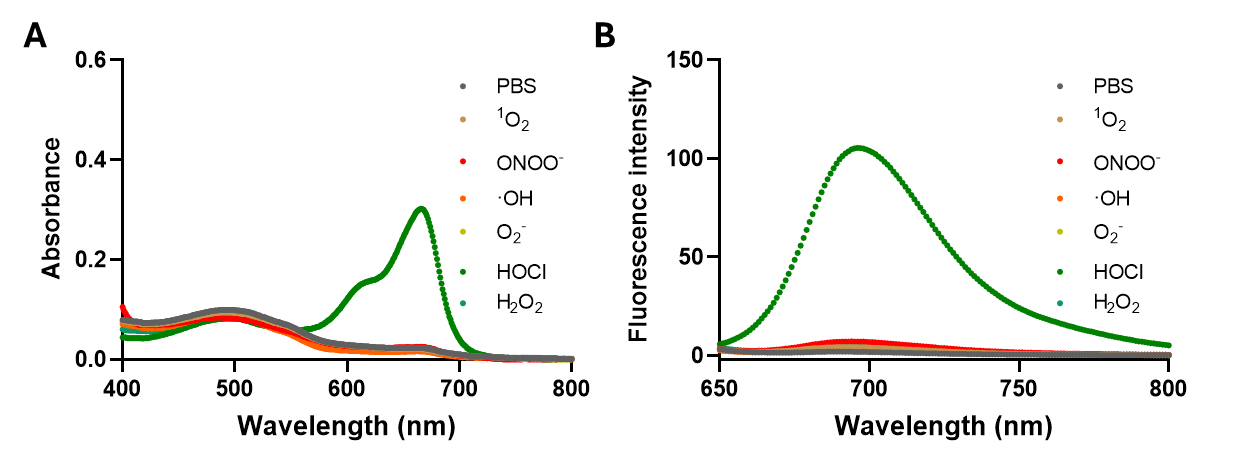


**Figure S8**. Characterization of MB-DOX in response to various ROS and RNS. (**A**) UV–vis absorbance and (**B**) fluorescence spectra of MB-DOX in the presence of 20 μM ROS (^1^O_2_, H_2_O_2_, O_2_^-^, HOCl and •OH) or RNS (ONOO^-^).


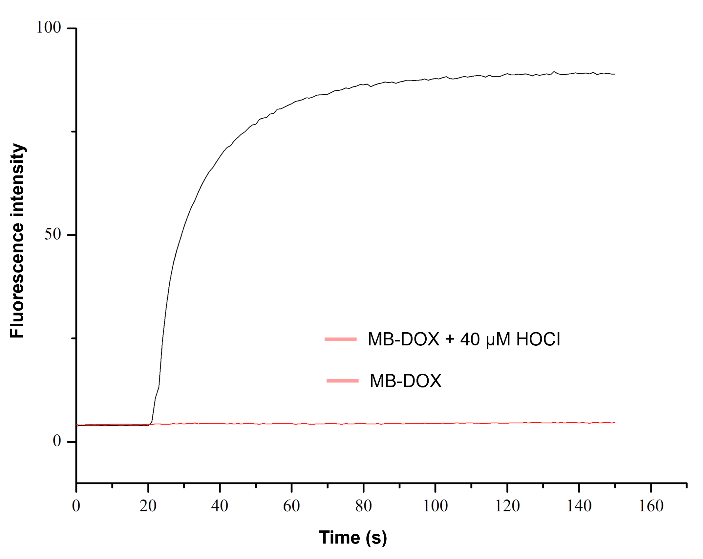


**Figure S9**. The temporal response of MB-DOX to 40 μM HOCl.


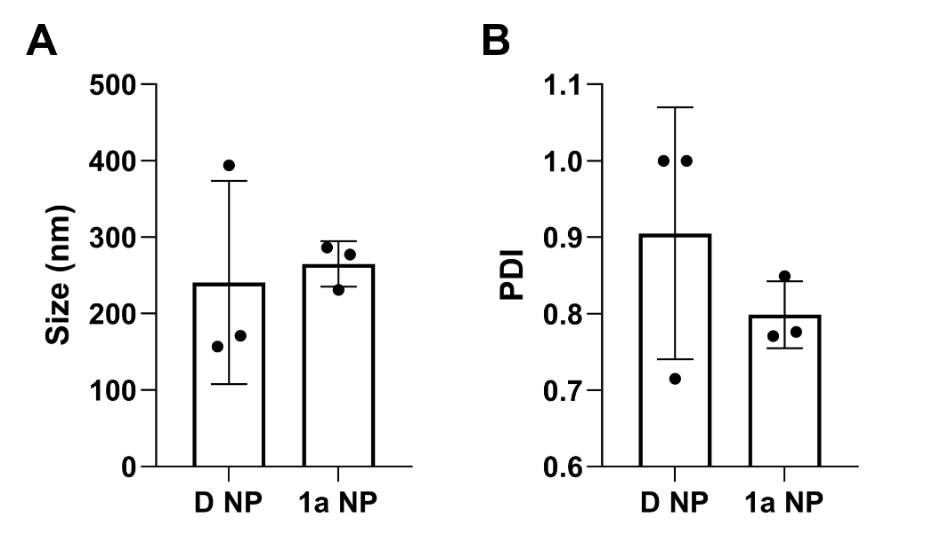


**Figure S10.** DLS analysis of D NP and 1a NP. (**A**) Particle size and (**B**) PDI of D NP and 1a NP. Data are presented as mean ± SD (n = 3).


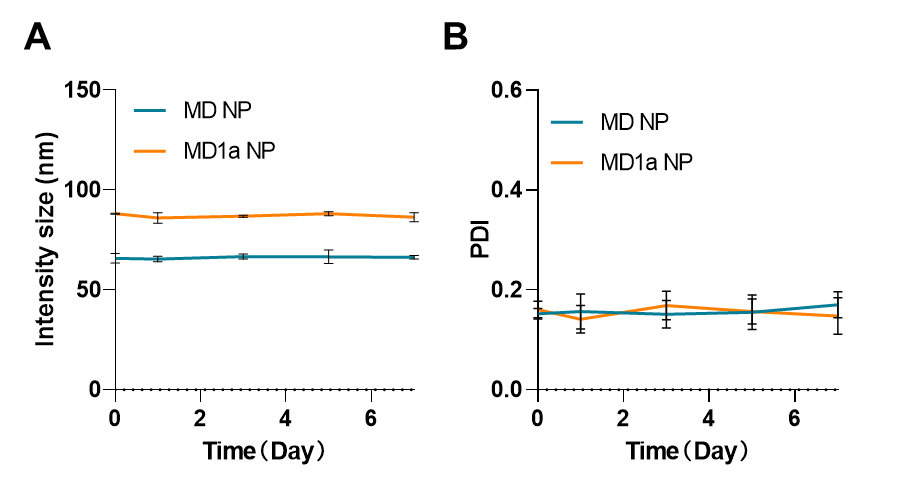


**Figure S11**. Changes in (**A**) intensity size and (**B**) PDI of MD NP and MD1a NP in PBS supplemented with 10% FBS over 1 week at 37°C. Data are represented as mean ± SD (n = 3).


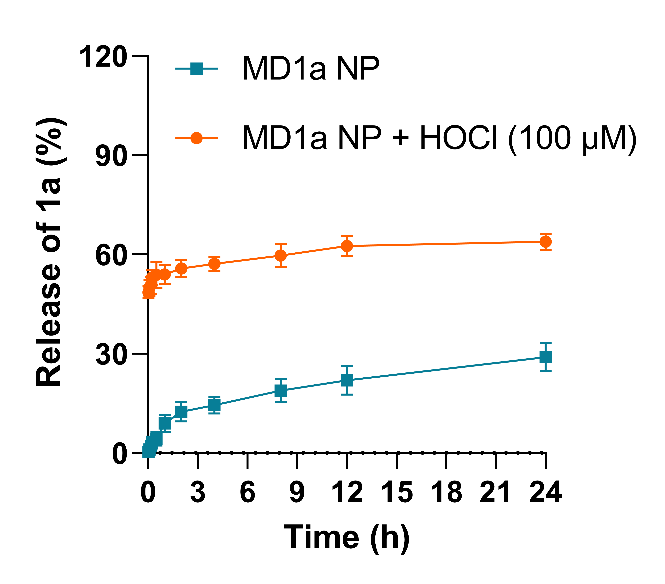


**Figure S12.** Cumulative release profiles of 1a from MD1a NP in the presence or absence of HOCl (100 μM). Data are presented as mean ± SD (n = 3).


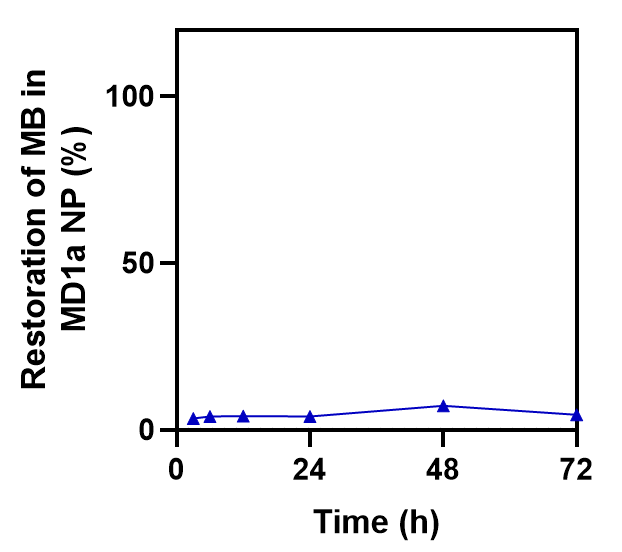


**Figure S13.** The restoration rate of MB within MD1a NP in PBS supplemented with 10% FBS at 37°C. Data are represented as mean ± SD (n = 3).


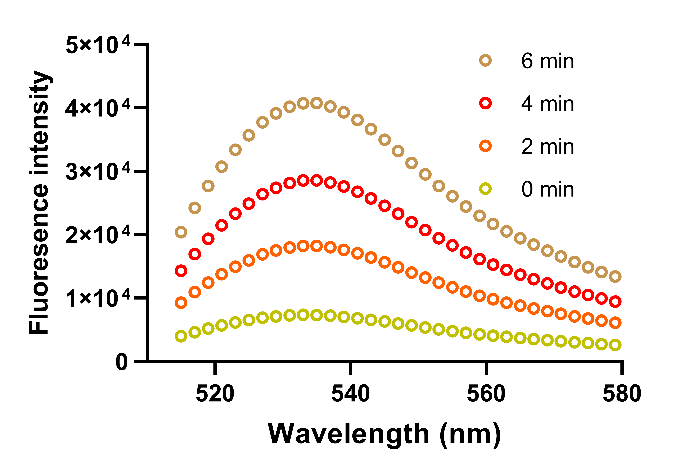


**Figure S14**. Fluorescence spectra of SOSG in HOCl (10 µM)-stimulated MD1a NP solution under NIR laser irradiation for 0, 2, 4, and 6 minutes.


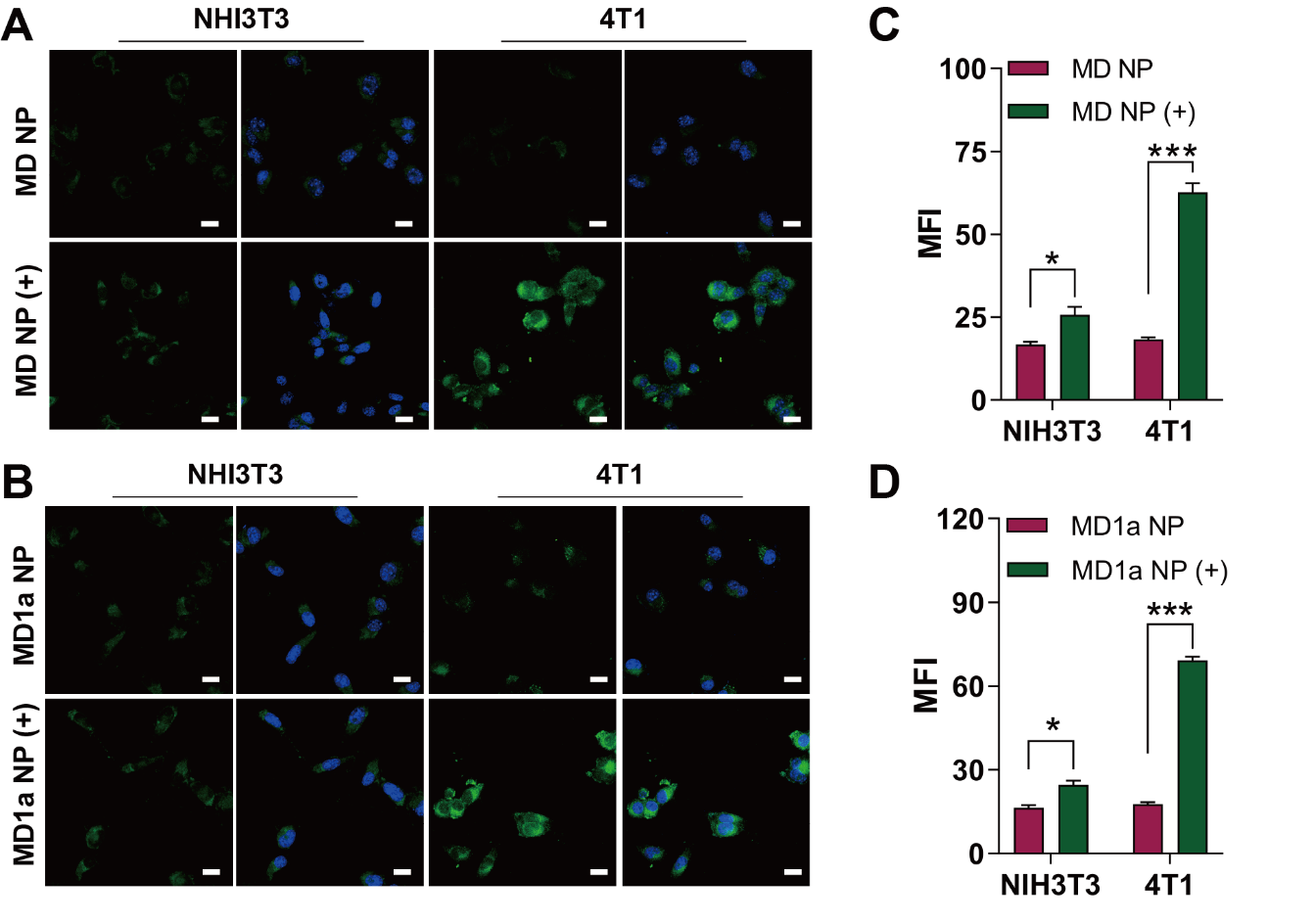


**Figure S15.** Representative CLSM images showing intracellular ROS levels in 4T1 and NIH3T3 cells treated with (**A**) MD NP and (**B**) MD1a NP, with or without NIR laser irradiation. Quantification of intracellular ROS levels in the (**C**) panel A and (**D**) in panel B (n = 3). Blue, DAPI; green, DCFH-DA fluorescence. Scale bar = 25 μm.


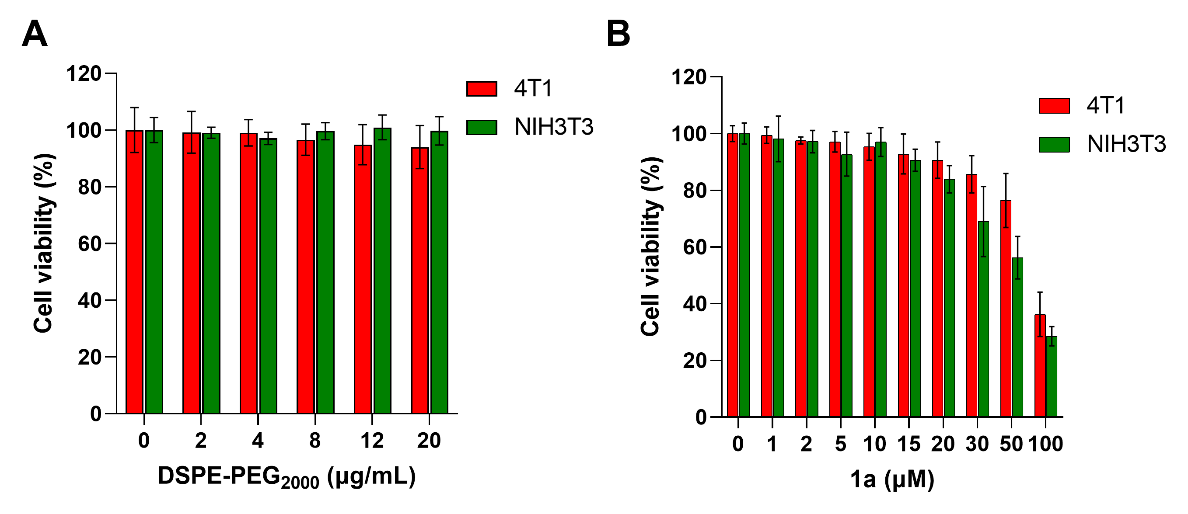


**Figure S16.** Cytotoxicity of (**A**) D NP and (**B**) free 1a in 4T1 and NIH3T3 cells at different concentrations. Data are presented as mean ± SD (n = 5).


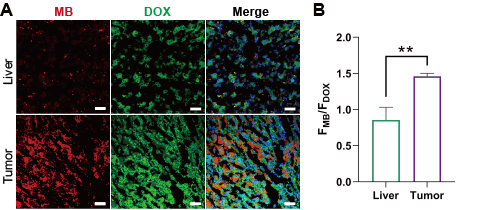


**Figure S17.** Co-release of MB and DOX in tumor and liver tissues. (**A**) Representative CLSM images of tumor and liver tissue sections from 4T1 tumor-bearing mice treated with MD1a NPs. Scale bar = 50 μm. (**B**) Quantitative analysis of the fluorescence intensity ratio of MB to DOX shown in panel A. Data are presented as mean ± SD (n = 3). **P < 0.01.


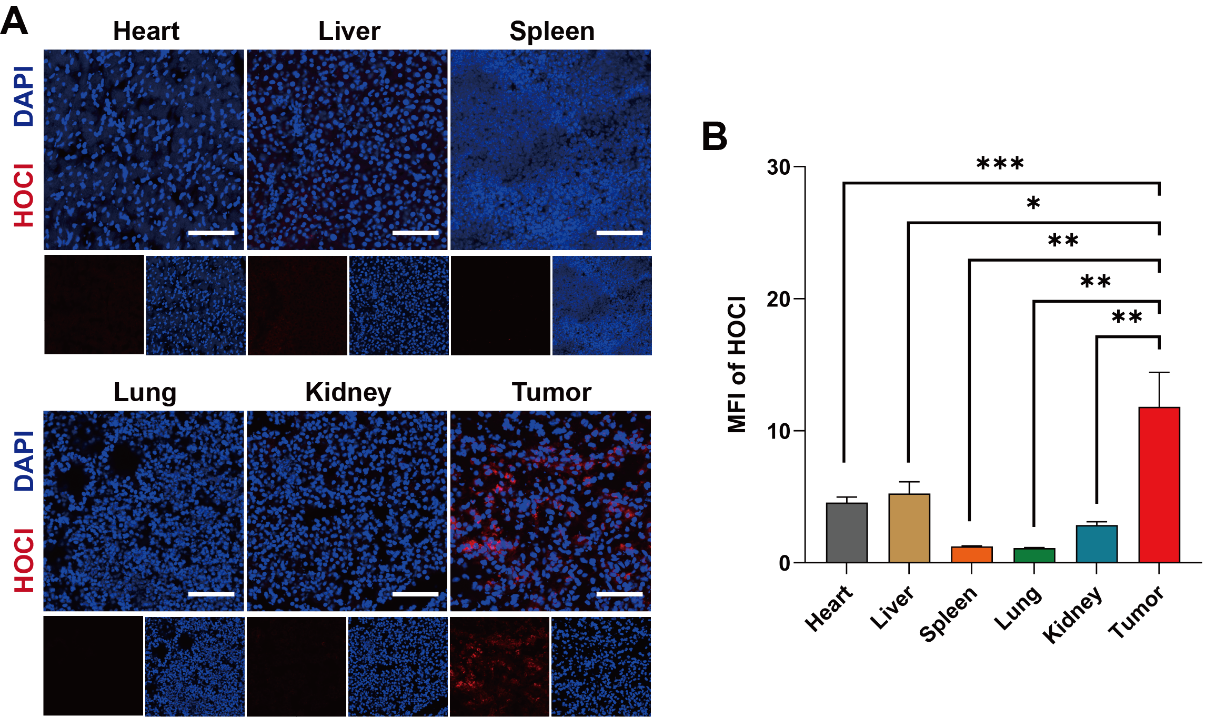


**Figure S18.** Evaluation of HOCl levels in different organs and tumor tissues. (**A**) Representative CLSM images showing HOCl levels in 4T1 cells under different treatments. Scale bar = 100 μm. (**B**) Quantification of HOCl fluorescence intensity shown in panel A. Data are presented as mean ± SD (n = 3). *P < 0.05, **P < 0.01, ***P < 0.001.


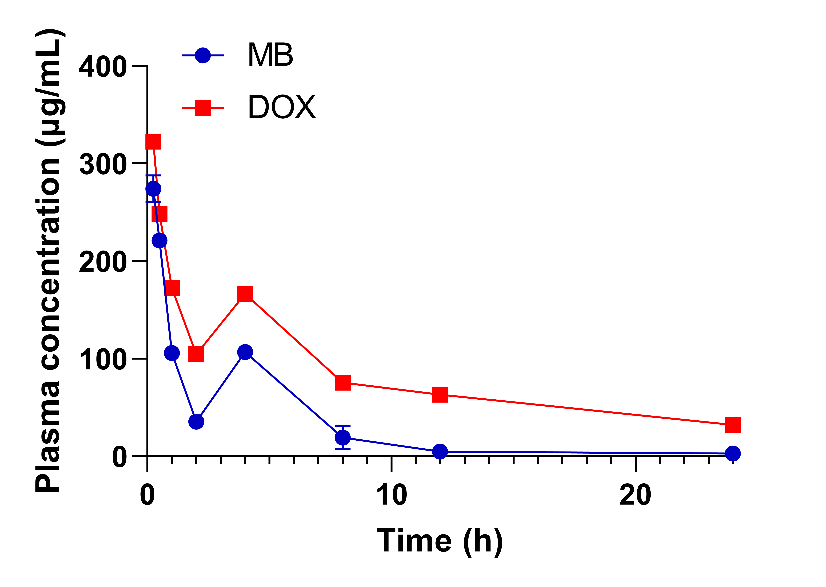


**Figure S19.** Plasma concentrations of MB and DOX after intravenous injection. Data are presented as mean ± SD (n = 3).


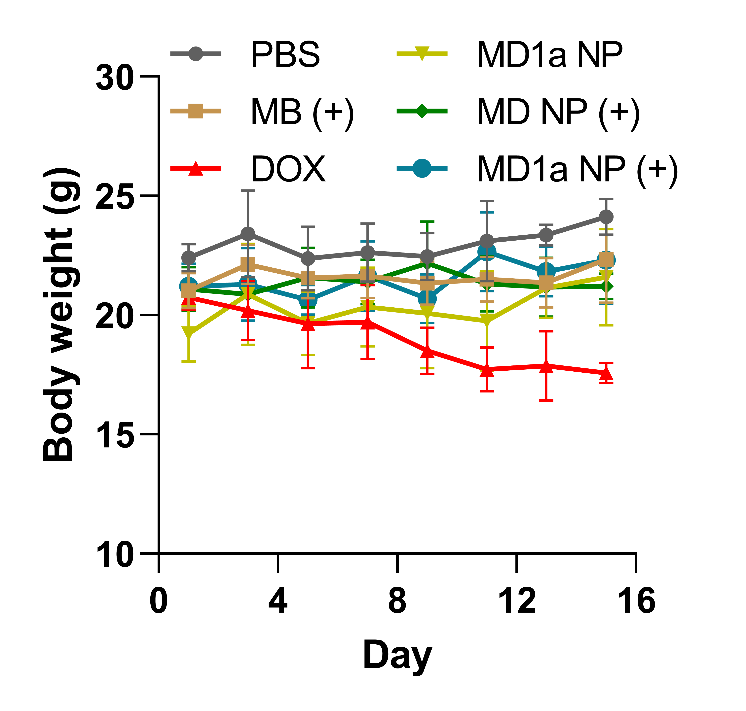


**Figure S20**. Body weight changes in 4T1 tumor-bearing mice following different treatments. Data are presented as mean ± SD (n = 5).


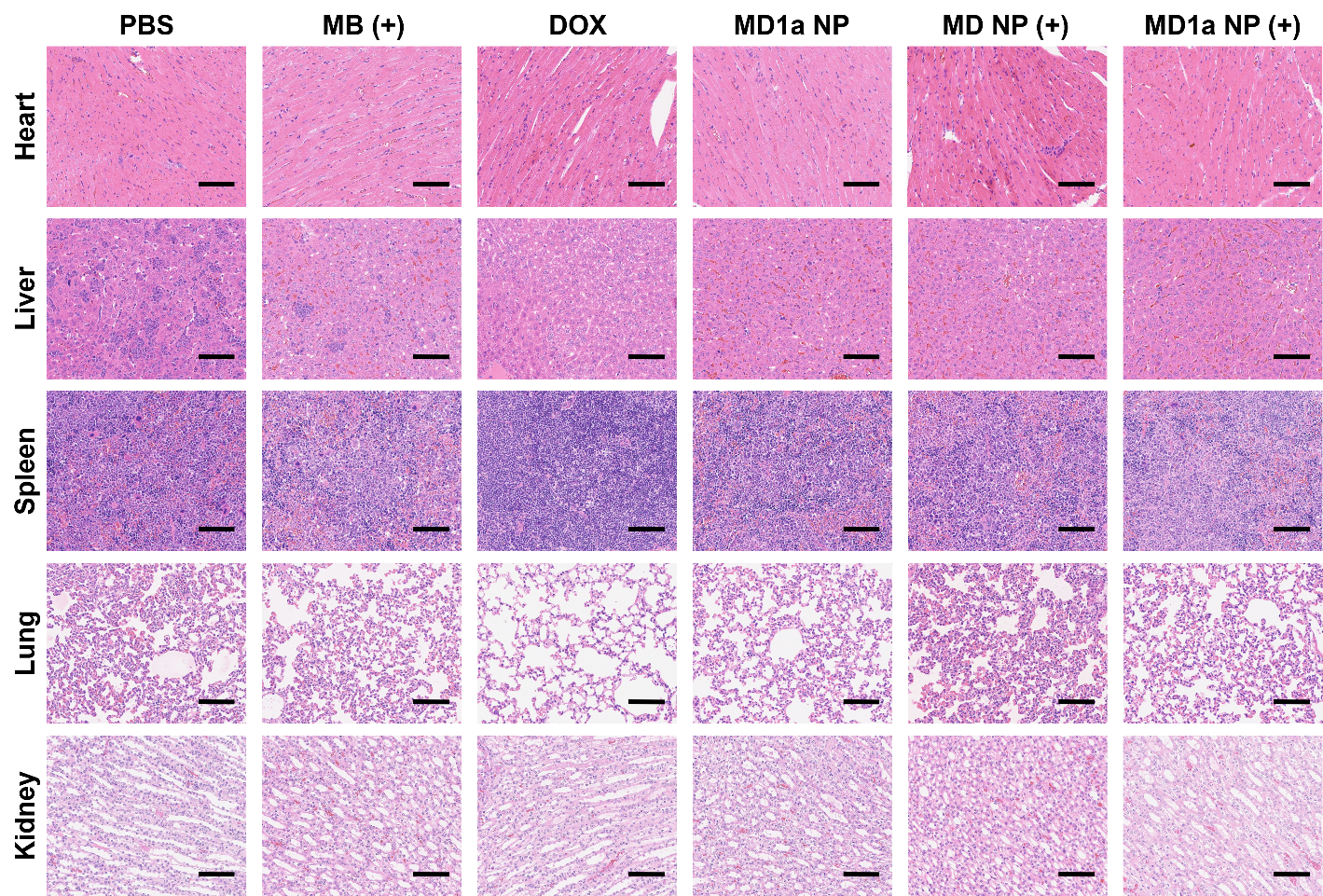


**Figure S21**. Representative H&E staining of major organs (heart, liver, spleen, lung, and kidney) from 4T1 tumor-bearing mice following different treatments. Bar=50 μm, n = 3.


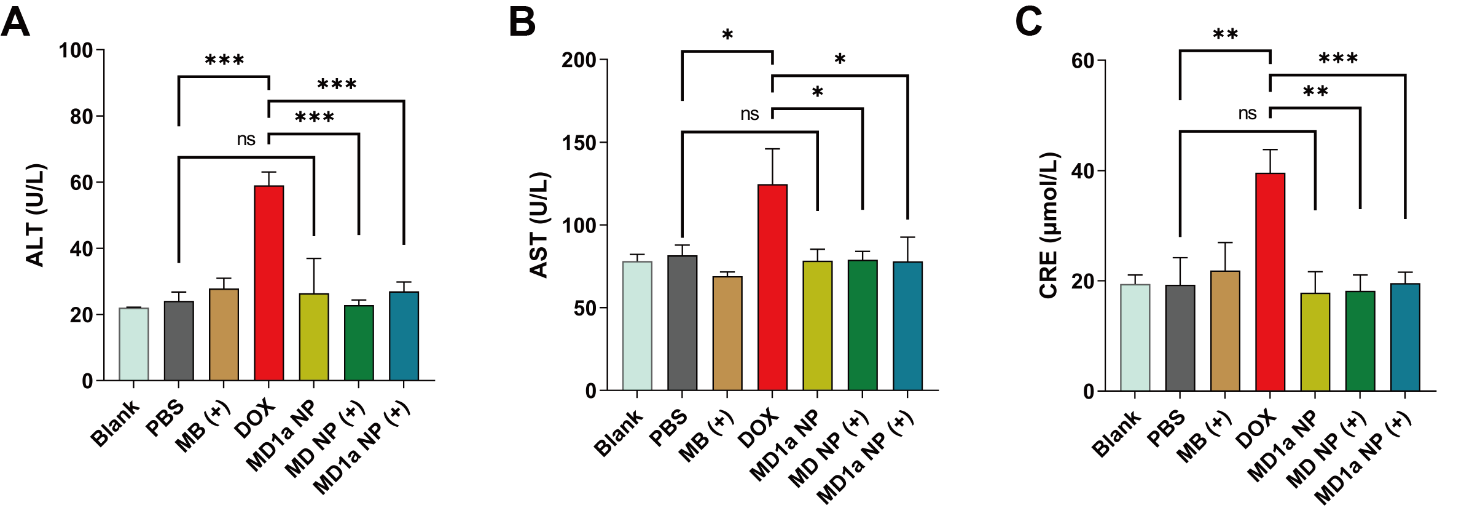


**Figure S22**. The levels of ALT, AST, and CRE in the serum of mice treated with different drug groups. Data are presented as mean ± SD (n = 3). ns, no significance, *p < 0.05; **p < 0.01, ***P < 0.001.


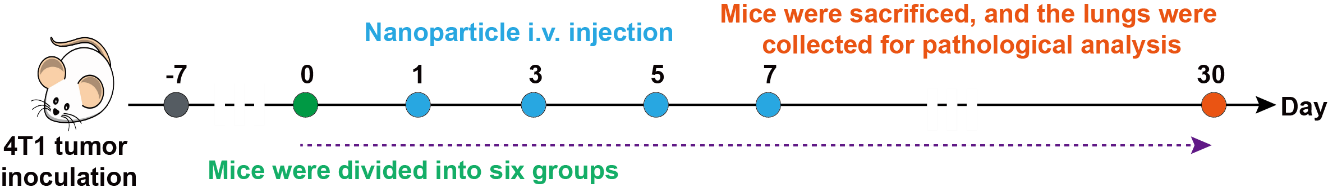


**Figure S23**. Illustration of the treatment procedure used to evaluate the prevention of lung metastasis.


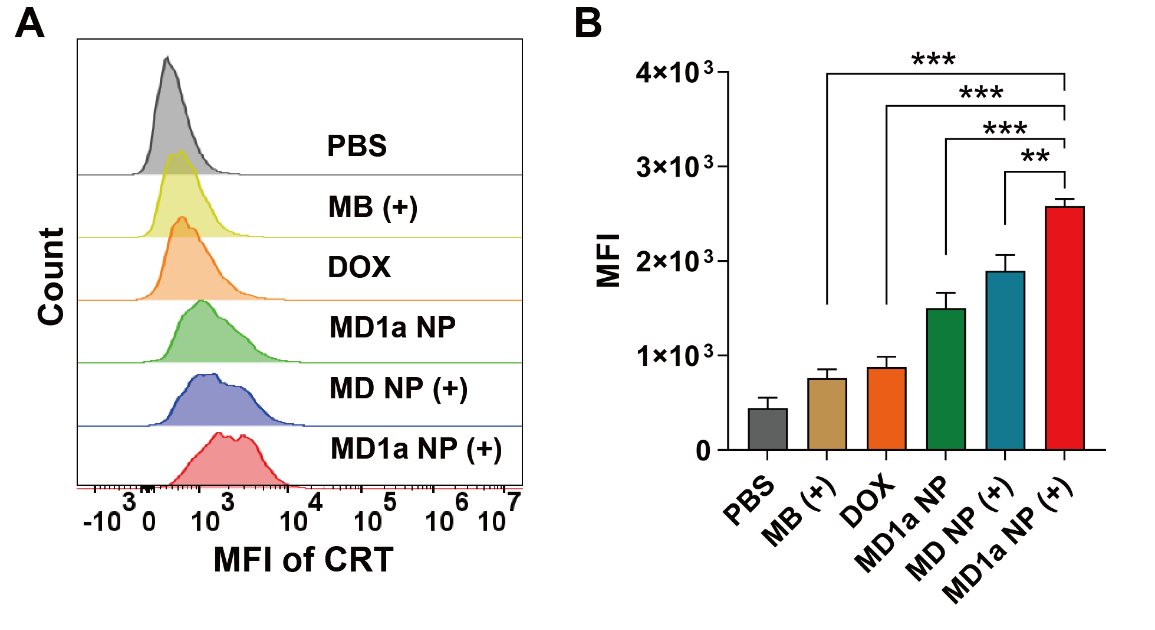


**Figure S24**. Evaluation of *in vivo* CRT exposure. (**A**) Representative flow cytometry plots and (**B**) quantification of CRT fluorescence intensity in 4T1 cells after different treatments. Data are presented as mean ± SD (n = 3). ***P < 0.001.


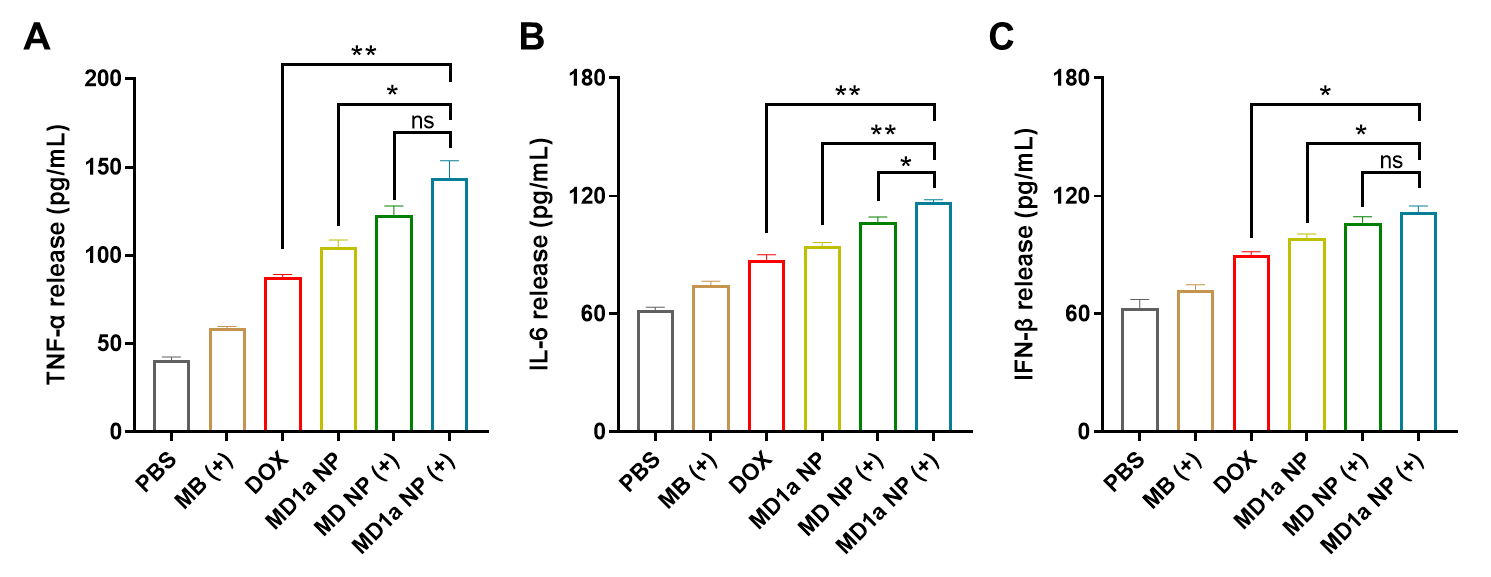


**Figure S25**. The levels of (**A**) TNF-α, (**B**) IL-6 and (**C**) IFN-β in the serum. Data are represented as mean ± SD (n = 3), ns, no significance, *p < 0.05; **p < 0.01.


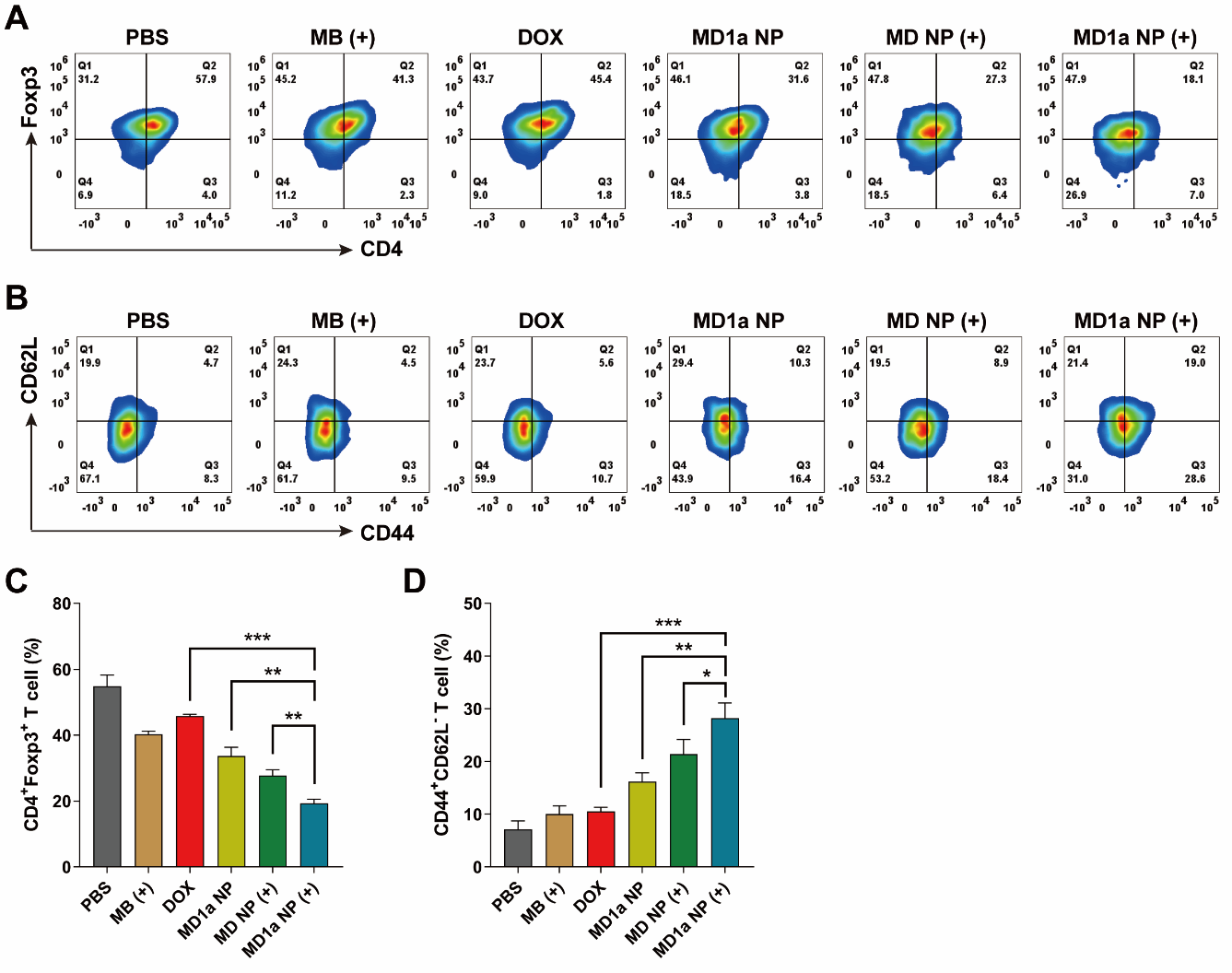


**Figure S26. Analysis of tumor-infiltrating Tregs and memory T cells.** (**A**) Representative flow cytometry plots of tumor-infiltrating Tregs (CD3⁺CD4⁺Foxp3⁺) in primary tumors under different treatments. (**B**) Representative flow cytometry plots of memory T cells (CD3⁺CD8⁺CD44⁺CD62L⁻) in the spleens of mice subjected to different treatments. Quantitative analysis of tumor-infiltrating (**C**) Tregs in primary tumors and (**D**) memory T cells in spleens from mice receiving different treatments. Data are presented as mean ± SD (n = 3). *P < 0.05, **P < 0.01, ***P < 0.001.

**
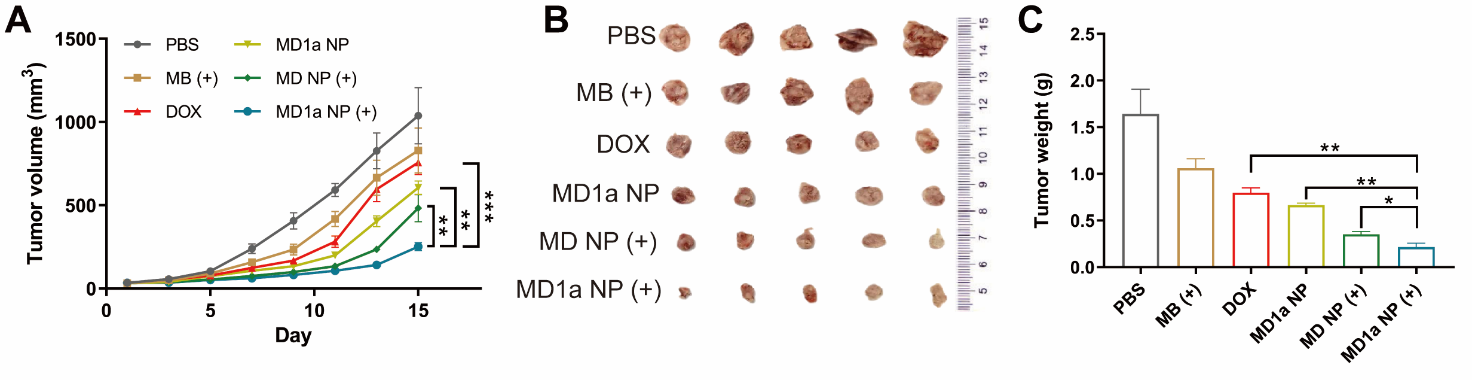
**

**Figure S27**. **Antitumor effects on primary tumors in a syngeneic bilateral 4T1 breast cancer model.** (**A**) Tumor growth curves, (**B**) tumor photographs, and (**C**) average tumor weights of primary tumors following different treatments (n = 5). Data are represented as mean ± SD, *p < 0.05; **p < 0.01; ***p < 0.001.
